# Lagrangian particle path formulation of multilayer shallow-water flows dynamically coupled to vessel motion

**DOI:** 10.1007/s10665-016-9893-3

**Published:** 2017-01-23

**Authors:** M. R. Turner, T. J. Bridges, H. Alemi Ardakani

**Affiliations:** Department of Mathematics, University of Surrey, Guildford, Surrey GU2 7XH UK

**Keywords:** Dynamic, Lagrangian particle path, Multilayer, Sloshing, Two-layer

## Abstract

The coupled motion—between multiple inviscid, incompressible, immiscible fluid layers in a rectangular vessel with a rigid lid and the vessel dynamics—is considered. The fluid layers are assumed to be thin and the shallow-water assumption is applied. The governing form of the Lagrangian functional in the Lagrangian particle path (LPP) framework is derived for an arbitrary number of layers, while the corresponding Hamiltonian is explicitly derived in the case of two- and three-layer fluids. The Hamiltonian formulation has nice properties for numerical simulations, and a fast, effective and symplectic numerical scheme is presented in the two- and three-layer cases, based upon the *implicit-midpoint rule*. Results of the simulations are compared with linear solutions and with the existing results of Alemi Ardakani et al. (J Fluid Struct 59:432–460, 2015) which were obtained using a finite volume approach in the Eulerian representation. The latter results are extended to non-Boussinesq regimes. The advantages and limitations of the LPP formulation and variational discretization are highlighted.

## Introduction

The Lagrangian and Eulerian descriptions of fluid motion are two viewpoints for representing fluid motion, with the Eulerian description being the most widely used in theoretical fluid dynamics. However, there are some settings where the Lagrangian particle path (LPP) description has advantages, one of which is shallow-water hydrodynamics. In the Eulerian form, the classical non-conservative shallow-water equations (SWEs) are

1.1 $$\begin{aligned} h_t + uh_x + hu_x = 0, \quad u_t + uu_x + gh_x = 0, \end{aligned}$$

where *h*(*x*, *t*) is the fluid depth, *u*(*x*, *t*) is the depth averaged horizontal velocity component, $g>0$ is the gravitational constant and the subscripts denote partial derivatives. Transforming to the LPP formulation gives

1.2 $$\begin{aligned} x_a \widehat{h}_\tau + \widehat{h}x_{a\tau } = 0, \quad x_{\tau \tau } + gx_a^{-1}\widehat{h}_a = 0, \end{aligned}$$

where $(a,\tau )$ are the label and time coordinate in the Lagrangian frame, fluid positions are represented by $x(a,\tau )$ and $\widehat{h}=\widehat{h}(a,\tau )$. The first equation in (1.2) can be integrated to $hx_a=\chi (a)$ where $\chi (a)$ is determined by the initial data, and substitution into the second equation gives

1.3 $$\begin{aligned} \frac{\partial ^2x}{\partial \tau ^2} + \frac{g}{x_a}\frac{\partial \ }{\partial a} \left( \frac{\chi }{x_a}\right) = 0 \,. \end{aligned}$$

Hence, the pair of equations (1.1) has been reduced to a single equation for *x*(*a*, *t*). Moreover the Eq. (1.3) is the Euler–Lagrange equation deduced, with fixed endpoint variations, from the Lagrangian functional

1.4 $$\begin{aligned} \mathscr {L}(x) = \int _{\tau _1}^{\tau _2}\int _{0}^{L} \left( \frac{1}{2}x_\tau ^2 - \frac{g}{2} \frac{\chi (a)}{x_a}\right) \chi (a) \mathrm{d}a\;\mathrm{d}\tau , \end{aligned}$$

where for definiteness $0<a<L$. The advantage of the transformation from (1.1) to (1.3) is that variational numerical schemes can be developed, by directly discretizing (1.4), which have excellent energy conservation properties. This energy conservation property is particularly important when the fluid motion is inside a vessel, and it is coupled to the vessel motion, as then it is of interest to accurately capture the energy partition between vessel and fluid. This strategy was used for simulating the dynamic coupling with a single-layer fluid in [1, 2].

The aim of this paper is to derive the LPP formulation to shallow water flows, with multiple layers of differing density, in a vessel with dynamic coupling, and use it as a basis for a variational formulation and numerical scheme. Although this generalisation is straightforward in principle, the variational formulation has complex subtleties due to the integration over different label spaces. Stewart and Dellar [3] successfully developed a variational formulation for shallow-water multilayer hydrodynamics by starting with a variational formulation for the full three-dimensional problem and reducing. The resulting variational principle for shallow water involves integration over each layer with respect to the local labels. With an aim to discretize the variational formulation, we modify the Stewart–Dellar formulation by introducing an explicit mapping between label spaces. Then all the integrations are over a single reference label space. Another addition to the variational formulation is that the multilayer shallow-water flow is dynamically coupled to the vessel motion. The theory will be developed in detail first for two-layers in Sect. 2 and then generalised to an arbitrary but finite number of layers in Sect. 4.

**Fig. 1:**
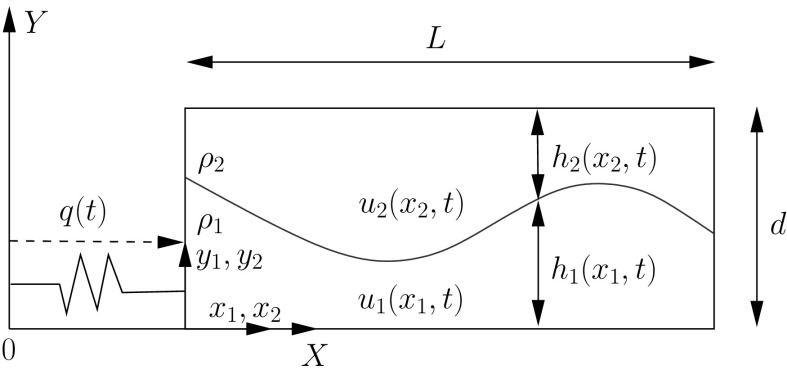
Schematic of the sloshing problem considered for the case of two-fluid layers. Here the vessel is constrained to move in the horizontal direction with displacement *q*(*t*). Here the displacement is determined by attaching the vessel to a nonlinear spring

A schematic of the problem of interest in the case of two layers is shown in Fig. 1. In Fig. 1, the fluid is coupled to a vessel undergoing horizontal motion only, as there is no vertical acceleration component.

This system is a model for the Offshore Wave Energy Ltd (OWEL) ocean wave energy converter [4]. The OWEL wave energy converter (WEC) is essentially a rectangular box, open at one end to allow waves to enter and, once they have entered the device, the interior sloshing causes the wave to grow pushing air through a turbine and generating electricity. This interior system is a two-layer flow of air and water confined between upper and lower surfaces. This paper considers a simplified model of the OWEL configuration by including two-layers of differing density, but in a closed vessel. In Fig. 1, the vessel displacement *q*(*t*) could be prescribed, i.e. the vessel is subjected to a prescribed horizontal time-dependent force, or it could be determined as part of the solution. In this paper, we consider the vessel to be attached to a nonlinear spring, and hence, the vessel motion is governed by a combination of the restoring force of the spring and the hydrodynamic force of the fluid on the side walls of the vessel. The moving vessel walls in turn create a force on the fluid causing it to move, thus the system undergoes complex coupled motions.

The literature on two-layer flows in open systems, with and without a rigid lid is vast ([5–8] to name a few), but in closed sloshing systems the literature is much more limited. The theoretical and experimental works of [9, 10] show excellent agreement for sloshing in a fixed rectangular tank with a rigid lid when a Lagrangian representation of the system is reduced to a system of ordinary differential equations with dissipative damping. Also, [11] examine two-layer sloshing in a forced vessel and derive a forced KdV equation when the layer thicknesses are comparable in size, an analysis of which shows that forcing induces chaos into the system through homoclinic tangles. The studies most relevant to the one in this paper examine the two-layer sloshing problem using a numerical scheme based upon a class of high resolution wave-propagating finite volume methods known as f-wave methods for both the forced [12] and the coupled problem [13]. This f-wave approach is very effective and can be readily be extended to multilayer systems [14] and systems with bottom topography [15], but [13] find the scheme is limited to layer density ratios of $\rho _2/\rho _1\gtrsim 0.7$, where $\rho _{1}$ and $\rho _2$ are the fluid densities in the lower and upper layers, respectively, due to a linear growth in the system constraint error. Therefore this approach is not able to model the interior workings of the OWEL WEC, where the air/water density ratio is $\rho _2/\rho _1\approx 10^{-3}$. The current paper formulates a simple numerical approach based upon a discretization of the LPP scheme, generalizing the numerics of [1] to two layers with nonlinear vessel motion. The LPP approach allows two-layer simulations with $\rho _2/\rho _1=10^{-3}$ to be produced.

The principal difficulty introduced by the rigid lid in the multilayer formulation is the Eulerian constraint

1.5 $$\begin{aligned} \sum _ih_i(x_i,t)=d, \end{aligned}$$

where ${h_i>0}$ and $x_i$ are the thickness of and fluid position in the $i\mathrm{th}$ layer, respectively, and the sum is over all the layers. In the LPP description, it is necessary to synchronise the position of the Lagrangian particles, otherwise the Eulerian constraint (1.5) will no longer hold at every spatial position. Here we overcome this problem by introducing layer mappings $\phi _i(a,\tau ):[0,L]\mapsto [0,L]$ such that the fluid position functions in layer *i* satisfy

$$\begin{aligned} x_i(\phi _i(a_1,\tau ),\tau )=x_1(a_1,\tau ),~~~\hbox {for}~~~i\ge 2, \end{aligned}$$

where $a_1$ is the Lagrangian label in layer 1, and $\tau$ is the Lagrangian time variable. The maps $\phi _i(a_1,\tau )$ are defined by these constraints. The maps $\phi _i$ become part of the variational formulation, and the integrals in the Lagrangian functionals are over the single reference space with labels $a_1$.

The paper is laid out as follows. In Sect. 2, the governing equations and variational principles for the two-layer rigid lid sloshing problem in the LPP description are presented. In Sect. 3 a variational discretization leading to a symplectic numerical integrator is introduced and simulations are presented. The results include validation of the scheme and extension of the numerical results into the non-Boussinesq regime. In Sect. 4, we demonstrate how the theory is extended to multilayer flows with a rigid upper lid and present simulations for the three-layer problem. Full details for the derivation of the governing three-layer equations is given in an appendix. Concluding remarks and discussion are presented in Sect. 5.

## Coupled equations with a two-layer fluid

In this section, we develop the equations for two-layer sloshing in a vessel with rectangular cross-section with a rigid lid coupled to horizontal vessel motion. A schematic of the problem is shown in Fig. 1.

The special case of two-layer flow is of interest for two reasons: Firstly, to simplify the analysis and make the derivation of the governing equations and solution procedure tractable and readable, and secondly because the underlying motivation for this work comes from the two-layer air-water flow inside the OWEL WEC. In Sect. 4 we document how the method presented in this section can be extended to incorporate multilayer shallow-water flow, and present simulation results for the case of three layers.

The rectangular vessel is a rigid body of length *L* and height *d* and we consider it filled with two immiscible, inviscid fluids of constant density $\rho _1$ and $\rho _2$ with $\rho _1>\rho _2$. The problem is assumed to be two-dimensional with the effect of the front and back faces of the vessel neglected. In what follows, the subscripts 1 and 2 denote the lower- and upper-layer variables respectively. There are two frames of reference in this problem, the inertial frame with coordinates $\mathbf{X}=(X,Y)$ and the body frame with coordinates $\mathbf{x}_i=(x_i,y_i)$ in each layer $i=1,2$. These coordinate systems are related via the time-dependent uniform translation *q*(*t*) in the $x_1-$direction, and in particular

$$\begin{aligned} X=x_1+q(t). \end{aligned}$$

In each layer, the thickness of the fluid $h_i(x_i,t)$ is assumed to be small such that the layer can be modelled using the shallow-water equations with a corresponding shallow-water velocity field $u_i(x_i,t)$. The rigid upper lid constrains the flow such that

2.1 $$\begin{aligned} h_1(x_1,t)+h_2(x_2,t)=d\,,\quad \hbox {when}~~~x_1=x_2\,. \end{aligned}$$

As we are considering a vessel with vertical side walls, we could consider the case where $x_1=x_2$ and thus only consider one spatial variable, but we leave our notation general for now, to highlight the interesting subtleties of the problem that arise when considering the Lagrangian form. The Eulerian form of the shallow-water mass and momentum equations in each layer in the body frame are

2.2 $$\begin{aligned}&\frac{\partial }{\partial t}(\rho _1h_1)+\frac{\partial }{\partial x_1}(\rho _1h_1u_1)=0, \end{aligned}$$

2.3 $$\begin{aligned}&\frac{\partial }{\partial t}(\rho _1u_1)+\frac{\partial }{\partial x_1}\left( \frac{1}{2}\rho _1u_1^2+\rho _1gh_1+\rho _2gh_2+p_s\right) =-\rho _1\ddot{q}, \end{aligned}$$

2.4 $$\begin{aligned}&\frac{\partial }{\partial t}(\rho _2h_2)+\frac{\partial }{\partial x_2}(\rho _2h_2u_2)=0, \end{aligned}$$

2.5 $$\begin{aligned}&\frac{\partial }{\partial t}(\rho _2u_2)+\frac{\partial }{\partial x_2}\left( \frac{1}{2}\rho _2u_2^2+\rho _2gd+p_s\right) =-\rho _2\ddot{q}, \end{aligned}$$

where $g>0$ is the gravitational acceleration constant, $p_s(x_2,t)$ is the unknown pressure at the rigid lid, and the over dots denote a full derivative with respect to *t* [13, 16]. The fluid in each layer must satisfy the no-penetration conditions on the vessel side walls, and hence, the boundary conditions are

$$\begin{aligned} u_i(0,t)=u_i(L,t)=0~~~\hbox {for}~~~i=1,2. \end{aligned}$$

The time-dependent motion of the vessel is not known *a priori* and is determined by a combination of a restoring force, such as a spring or a pendulum [17] and a hydrodynamic force exerted on the vessel side walls by the sloshing fluid. We assume that the vessel is connected to the spatial origin by a nonlinear spring, and hence, the vessel motion is governed by

2.6 $$\begin{aligned} \frac{\mathrm{d}}{\mathrm{d}t}\left[ \int _0^L\rho _1h_1u_1\,\mathrm{d}x_1+\int _0^L\rho _2h_2u_2\,\mathrm{d}x_2+\left( m_f^{(1)}+m_f^{(2)}+m_v\right) \dot{q}\right] +\nu _1q-\nu _2q^3=0, \end{aligned}$$

where $\nu _1$ and $\nu _2$ are constant spring coefficients and $m_f^{(i)}=\int _0^L\rho _ih_i(x_i,t)\,\mathrm{d}x_i~~i=1,~2$ is the fluid mass in the $i\mathrm{th}$ layer. Here the integrals on the LHS of (2.6) denote the hydrodynamic force contribution of each layer to the vessel motion.

Equations (2.1)–(2.6) can be derived from an *Eulerian variational principle* by considering variations to the Lagrangian functional

2.7 $$\begin{aligned} \mathscr {L}=\int _{t_1}^{t_2}\left( \int _0^L L_1 \,\mathrm{d}x_1+\int _0^L L_2 \,\mathrm{d}x_2\right) \mathrm{d}t+\int _{t_1}^{t_2}\left( \frac{1}{2}m_v\dot{q}^2-\frac{1}{2}\nu _1q^2+\frac{1}{4}\nu _2q^4\right) \,\mathrm{d}t, \end{aligned}$$

where

$$\begin{aligned} L_1= & {} \frac{1}{2}\rho _1h_1\left( u_1+\dot{q}\right) ^2-\frac{1}{2}\rho _1gh_1^2+\rho _1f_1\left( h_{1t}+(h_1u_1)_{x_1}\right) -\rho _2gh_1h_2,\\ L_2= & {} \frac{1}{2}\rho _2h_2\left( u_2+\dot{q}\right) ^2-\frac{1}{2}\rho _2gh_2^2+\rho _2f_2\left( h_{2t}+(h_2u_2)_{x_2}\right) -p_s(h_1+h_2-d). \end{aligned}$$

Here $p_s(x_2,t)$ enters as a Lagrange multiplier for the constraint, and $f_1(x_1,t)$ and $f_2(x_2,t)$ are additional Lagrange multipliers for the mass conservation equations (2.2) and (2.4). The Lagrangian in (2.7) is comprised of three Lagrangian functionals, one for the dry vessel and one for each fluid layer, as discussed in Sect. 4.6 of [3], where the term $-\rho _2gh_1h_2$ in $L_1$ is identified as the work done on the upper surface of the lower layer by the layer above, and the terms proportional to $(u_i+\dot{q})^2$ couple the fluid motion to the vessel motion. One feature of the Lagrangian (2.7) is that the additional work term in $L_1$, $-\rho _2gh_1h_2$, is a function of $x_1,~x_2$ and *t*, but the integral, as written above, is over $x_1$, moreover, as discussed earlier, the Eulerian constraint $h_1(x_1,t)+h_2(x_2,t)=d$ has to hold for $x_1=x_2$. Both of these issues are overcome in Sect. 2.1 by introducing the constraint that $x_1=x_2$ into (2.7) and formulating the problem in terms of the lower layer coordinate only.

The shallow-water equations (2.2)–(2.5) could be solved numerically via some implicit shallow-water numerical scheme, with the vessel equation (2.6) integrated via standard fourth-order Runge–Kutta integration. However, this scheme would not necessarily have good energy conservation properties. Hence, in order to model the long-time oscillatory behaviour of the system, we construct a Hamiltonian formulation of the system in order to utilise geometric integration schemes. We do this by transforming the above Eulerian variational formulation to an LPP Lagrangian variational formulation.

### LPP description

#### Lagrangian variational formulation

To transform the Eulerian shallow-water equations into a LPP formulation, we need to consider mappings from the Lagrangian particle labels and Lagrangian time $(a_i,\tau )$ in each layer to the corresponding Cartesian coordinates and Eulerian time $(x_i,t)$. This again demonstrates another peculiar feature of the problem, because there is no guarantee that for all $\tau$, $x_1(a_1,\tau )=x_2(a_2,\tau )$ which we require so as to satisfy the Eulerian constraint (2.1). The approach to overcome this problem is to link the two LPP labels in each layer via $a_2=\phi (a_1,\tau )$ where $\phi (a_1,\tau )$ is an unknown map to be determined. In the subsequent analysis, we shall drop the subscript 1 from the Lagrangian label $a_1$ with the understanding that this is the label in the lower layer, and our primary reference label.

To derive the LPP formulation of the problem, consider the mapping

2.8 $$\begin{aligned} (\tau ,a)\longmapsto (t,x_1(a,\tau ))~~~\hbox {with}~~~0\le a\le L,~~~\tau \ge 0, \end{aligned}$$

with the constraint that in the upper layer

2.9 $$\begin{aligned} x_1(a,\tau )=x_2(\phi (a,\tau ),\tau ). \end{aligned}$$

We assume that the mapping is non-degenerate $({\partial x_1}/{\partial a}\ne 0)$ and thus the derivatives in (2.2) and (2.3) map to

2.10 $$\begin{aligned} \frac{\partial }{\partial x_1}\longmapsto \frac{1}{x_{1a}}\frac{\partial }{\partial a},~~~\hbox {and}~~~\frac{\partial }{\partial t}\longmapsto \frac{\partial }{\partial \tau }-\frac{x_{1\tau }}{x_{1a}}\frac{\partial }{\partial a}, \end{aligned}$$

where here the subscripts *a* and $\tau$ denote partial derivatives. Because we have assumed the constraint (2.9) the derivatives in (2.4) and (2.5) map in the same way as in (2.10), but we can show this formally. From the form of $x_2$ in (2.9), the derivatives in the LPP setting map on to

$$\begin{aligned} \frac{\partial }{\partial x_2}\longmapsto \frac{1}{x_{2\phi }\phi _a}\frac{\partial }{\partial a},~~~\hbox {and}~~~\frac{\partial }{\partial t}\longmapsto \frac{\partial }{\partial \tau }-\frac{(x_{2\tau }+x_{2\phi }\phi _\tau )}{x_{2\phi }\phi _a}\frac{\partial }{\partial a}. \end{aligned}$$

But we note from (2.9) that

$$\begin{aligned} x_{1a}=x_{2\phi }\phi _a,~~~\hbox {and}~~~x_{1\tau }=x_{2\tau }+x_{2\phi }\phi _\tau , \end{aligned}$$

and thus the derivatives in the upper layer also map as in (2.10) as noted above.

Under this LPP transformation, the fluid equations in each layer, (2.2)–(2.5), transform to

2.11 $$\begin{aligned}&(\widehat{h}_1x_{1a})_\tau =0, \end{aligned}$$

2.12 $$\begin{aligned}&x_{1\tau \tau }+g\left( 1-\frac{\rho _2}{\rho _1}\right) \frac{\widehat{h}_{1a}}{x_{1a}}+\frac{1}{\rho _1 x_{1a}}p_{sa}=-q_{\tau \tau }, \end{aligned}$$

2.13 $$\begin{aligned}&x_{1a}\widehat{h}_{2\tau }-x_{1\tau }\widehat{h}_{2a}+(\widehat{h}_2x_{2\tau })_a=0, \end{aligned}$$

2.14 $$\begin{aligned}&x_{2\tau \tau }+\frac{1}{\rho _2}\frac{p_{sa}}{x_{1a}}=-q_{\tau \tau }, \end{aligned}$$

where the constraint (2.1) has been used to remove $\widehat{h}_2=d-\widehat{h}_1$ from (2.12). Equation (2.11) implies that

2.15 $$\begin{aligned} \widehat{h}_1=\frac{\chi (a)}{x_{1a}},~~~\hbox {where}~~~\chi (a)=\widehat{h}_1x_{1a}\Big |_{\tau =0}, \end{aligned}$$

while adding (2.11) to (2.13) and using (2.1) lead to the mass flux condition

$$\begin{aligned} (\widehat{h}_1x_{1\tau }+\widehat{h}_2x_{2\tau })_a=0, \end{aligned}$$

or

2.16 $$\begin{aligned} \widehat{h}_1x_{1\tau }+\widehat{h}_2x_{2\tau }=0, \end{aligned}$$

after integrating and using the side wall boundary conditions to fix the time-dependent integration function. Eliminating $p_s$ between (2.12) and (2.14) and using (2.16) to eliminate $x_{2\tau }$, (2.1) to eliminate $\widehat{h}_2$ and (2.15) to eliminate $\widehat{h}_1$ lead to a PDE in $x_1(a,\tau )$ and $q(\tau )$ only,

2.17 $$\begin{aligned}&x_{1\tau \tau }-\frac{\rho _2}{\rho _1}\left[ -\frac{\chi x_{1\tau \tau }}{dx_{1a}-\chi }+\frac{2d\chi x_{1\tau }x_{1a\tau }}{(dx_{1a}-\chi )^2}+\frac{d^2x_{1\tau }^2x_{1a}^2}{(dx_{1a}-\chi )^3}\left( \frac{\chi }{x_{1a}}\right) _a\right] \nonumber \\&\quad +\,g\left( 1-\frac{\rho _2}{\rho _1}\right) \frac{1}{x_{1a}}\left( \frac{\chi }{x_{1a}}\right) _a=-\left( 1-\frac{\rho _2}{\rho _1}\right) q_{\tau \tau }, \end{aligned}$$

which is coupled to vessel equation (2.6) which in the LPP description is

2.18 $$\begin{aligned} \frac{\mathrm{d}^2}{\mathrm{d}\tau ^2}\left[ (\rho _1-\rho _2)\int _0^L\chi x_1\,\mathrm{d}a+\left( m_f^{(1)}+m_f^{(2)}+m_v\right) q\right] +\nu _1q-\nu _2q^3=0. \end{aligned}$$

Equation (2.17) is the analogous form of the one-layer, unforced equation (1.3).

The pair of equations (2.17) and (2.18) can also be determined by a variational approach from the Lagrangian (2.7) converted into the LPP description. We directly impose the constraints, and use (2.15) and (2.16) to write the Lagrangian solely in terms of $x_1$ and *q*, which takes the form

2.19 $$\begin{aligned} \mathscr {L}(x_1,q)=\int _{\tau _1}^{\tau _2}\int _0^L \widetilde{L}\,\mathrm{d}a\mathrm{d}\tau +\int _{\tau _1}^{\tau _2}\left( \frac{1}{2}m_vq_\tau ^2-\frac{1}{2}\nu _1q^2+\frac{1}{4}\nu _2q^4\right) \,\mathrm{d}\tau , \end{aligned}$$

where

$$\begin{aligned} \widetilde{L}=\frac{1}{2}\rho _1\chi \left( x_{1\tau }+q_\tau \right) ^2+\frac{1}{2}\rho _2(dx_{1a}-\chi )\left( -\frac{\chi x_{1\tau }}{dx_{1a}-\chi }+q_\tau \right) ^2-\frac{1}{2}g(\rho _1-\rho _2)\frac{\chi ^2}{x_{1a}}-\frac{1}{2}\rho _2gd^2x_{1a}. \end{aligned}$$

Taking variations, with fixed endpoints, with respect to $x_1$ and *q* (e.g. writing $q=q+\delta q$ with $\delta q(\tau _1)=\delta q(\tau _2)=0$) leads to (2.17) and (2.18) respectively.

Note that in the case $\rho _2=0$ (with $\nu _2=0$), (2.19) reduces to the one-layer coupled Lagrangian given in [1], i.e. in this case the fluid does not feel the effect of the rigid lid.

#### Hamiltonian formulation

The coupled Lagrangian system (2.19) can also be written in terms of a Hamiltonian functional with canonical variables $(x_1,q,w,p)$. The momentum variables are

$$\begin{aligned}&w(a,\tau )=\frac{1}{\chi }\frac{\delta \mathscr {L}}{\delta x_{1\tau }}=\rho _1(x_{1\tau }+q_\tau )-\rho _2\left( -\frac{\chi x_{1\tau }}{dx_{1a}-\chi }+q_\tau \right) ,\\&p(\tau )=\frac{\delta \mathscr {L}}{\delta q_{\tau }}=\int _0^L\left[ \rho _1\chi (x_{1\tau }+q_\tau )+\rho _2(dx_{1a}-\chi )\left( -\frac{\chi x_{1\tau }}{dx_{1a}-\chi }+q_\tau \right) \right] \,\mathrm{d}a+m_vq_\tau , \end{aligned}$$

which can be written in the more convenient form

2.20 $$\begin{aligned}&w(a,\tau )=\frac{\chi (\rho _1-\rho _2)}{B}x_{1\tau }+(\rho _1-\rho _2)q_{\tau }, \end{aligned}$$

2.21 $$\begin{aligned}&p(\tau )=Aq_\tau +\int _0^LBw\,\mathrm{d}a, \end{aligned}$$

where

$$\begin{aligned} A(\tau )= & {} m_v+m_f^{(1)}+m_f^{(2)}-(\rho _1-\rho _2)\int _0^LB(a,\tau )\,\mathrm{d}a,\\ B(a,\tau )= & {} \frac{\chi (dx_{1a}-\chi )(\rho _1-\rho _2)}{\rho _1(dx_{1a}-\chi )+\rho _2\chi }. \end{aligned}$$

The Hamiltonian can then be formed by taking the Legendre transformation of the Lagrangian (2.19). The Hamiltonian functional is given by

2.22 $$\begin{aligned} \mathscr {H}(x_1,q,w,p)= & {} \frac{1}{2(\rho _1-\rho _2)}\int _0^L Bw^2\,\mathrm{d}a +\frac{1}{2A}\left( p-I\right) ^2\nonumber \\&+\;\frac{1}{2}(\rho _1-\rho _2)g\int _0^L\frac{\chi ^2}{x_{1a}}\,\mathrm{d}a+\frac{1}{2}\rho _2gd^2L+\frac{1}{2}\nu _1q^2-\frac{1}{4}\nu _2q^4, \end{aligned}$$

where $I=\int _0^LBw\,\mathrm{d}a$, and the governing form of Hamilton’s equations are

2.23 $$\begin{aligned} -w_\tau =\frac{1}{\chi }\frac{\delta \mathscr {H}}{\delta x}= & {} g(\rho _1-\rho _2)\frac{1}{2\chi }\left( \frac{\chi ^2}{x_{1a}^2}\right) _a-\frac{d\rho _2}{\chi }\Bigg [\frac{1}{2}\left( \frac{\chi ^2w^2}{\Gamma ^2}\right) _a\nonumber \\&\quad -\,\frac{\left( p-I\right) }{A}(\rho _1-\rho _2)\left( \frac{\chi ^2w}{\Gamma ^2}\right) _a+\frac{\left( p-I\right) ^2}{2A^2}(\rho _1-\rho _2)^2\left( \frac{\chi ^2}{\Gamma ^2}\right) _a\Bigg ], \end{aligned}$$

2.24 $$\begin{aligned}&-p_\tau =\frac{\delta \mathscr {H}}{\delta q}=\nu _1q-\nu _2q^3, \end{aligned}$$

2.25 $$\begin{aligned}&x_\tau =\frac{1}{\chi }\frac{\delta \mathscr {H}}{\delta w}=\frac{Bw}{\chi (\rho _1-\rho _2)}-\frac{B}{\chi }\frac{\left( p-I\right) }{A}, \end{aligned}$$

2.26 $$\begin{aligned}&q_\tau =\frac{\delta \mathscr {H}}{\delta p}=\frac{\left( p-I\right) }{A}, \end{aligned}$$

where

$$\begin{aligned} \Gamma (a,\tau )=\rho _1(dx_{1a}-\chi )+\rho _2\chi . \end{aligned}$$

Here, as in [1], the gradient of $\mathscr {H}$ is taken with respect to the weighted inner product such that

$$\begin{aligned} \left\langle \left\langle \nabla \mathscr {H},\delta Z \right\rangle \right\rangle =\int _0^L\left( \frac{\delta \mathscr {H}}{\delta x_1}\delta x_1+\frac{\delta \mathscr {H}}{\delta w}\delta w\right) \chi \,\mathrm{d}a+\frac{\delta \mathscr {H}}{\delta q}\delta q+\frac{\delta \mathscr {H}}{\delta p}\delta p, \end{aligned}$$

where $$Z=(x_1,q,w,p)$$.

The form of (2.23) is equivalent to that in (2.17), which was derived directly from the Eulerian form of the equations. This equivalence is shown in Appendix 1.

### Linear solutions to LPP problem

The linear solution of the two-layer shallow-water sloshing problem with a rigid lid in the Eulerian framework has been discussed in detail in [13]. However, the form of this linear solution in the LPP framework would be desirable in order to use it as an initial condition when numerically integrating Hamilton’s equations so to validate the scheme. Hence, we briefly outline the linear solution procedure here.

We seek a linear solution to (2.17) about a quiescent fluid with the lower layer of mean thickness $h_1^0$ of the form

$$\begin{aligned} x_1(a,\tau )=a+X_1(a,\tau ),~~~\hbox {and}~~~\widehat{h}_1(a,\tau )=h_1^0+H_1(a,\tau ), \end{aligned}$$

where we assume$|X_1|\ll 1$, $|H_1|\ll 1$ and $|q|\ll 1$. Substituting these expressions into $\widehat{h}_1x_{1a}=\chi (a)$ leads to

$$\begin{aligned} \chi (a)=h_1^0,~~~\hbox {and}~~~H_1+h_1^0X_{1a}=0, \end{aligned}$$

while substitution into (2.17) leads to

2.27 $$\begin{aligned} X_{1\tau \tau }-\frac{h_1^0h_2^0g(\rho _1-\rho _2)}{\rho _1h_2^0+\rho _2h_1^0}X_{1aa}=-\frac{h_2^0(\rho _1-\rho _2)}{\rho _1h_2^0+\rho _2h_1^0}q_{\tau \tau }, \end{aligned}$$

where $h_2^0=d-h_1^0=d-\chi$ is the mean thickness of the fluid in the upper layer.

Seeking a harmonic solution of these equations with frequency $\omega$ emits the separable variable forms

2.28 $$\begin{aligned} X_1(a,\tau )=\widehat{X}_1(a)\cos (\omega \tau ),~~~q(\tau )=\widehat{q}\cos (\omega \tau ),~~~H_1(a,\tau )=\widehat{H}_1(a)\cos (\omega \tau ), \end{aligned}$$

which transforms (2.27) to

2.29 $$\begin{aligned} \widehat{X}_{1aa}+\alpha ^2\widehat{X}_1+\beta \widehat{q}=0, \end{aligned}$$

where

$$\begin{aligned} \alpha ^2=\frac{\rho _1h_2^0+\rho _2h_1^0}{h_1^0h_2^0g(\rho _1-\rho _2)}\omega ^2,~~~\hbox {and}~~~\beta =\frac{\omega ^2}{h_1^0g}. \end{aligned}$$

The general solution to (2.29) satisfying $x_1(0,\tau )=0$ ($\widehat{X}_1(0)=0$) is

2.30 $$\begin{aligned} \widehat{X}_1=\frac{\beta \widehat{q}}{\alpha ^2}\left[ \cos (\alpha a)-1\right] +\gamma \sin (\alpha a), \end{aligned}$$

where $\gamma$ is an as yet undetermined constant, and when we satisfy $x_{1}(L,\tau )=L$ ($\widehat{X}_1(L)=0$) we find the relation on $\gamma$ and $\widehat{q}$ that

2.31 $$\begin{aligned} 0=\gamma \sin \left( \frac{1}{2}\alpha L\right) \cos \left( \frac{1}{2}\alpha L\right) -\frac{\beta \widehat{q}}{\alpha ^2}\sin ^2\left( \frac{1}{2}\alpha L\right) . \end{aligned}$$

The linear form of the vessel equation (2.18) upon substitution of (2.28), leads to

$$\begin{aligned} -(\rho _1-\rho _2)h_1^0\omega ^2\int _0^L\widehat{X}_1\,\mathrm{d}a-\widehat{M}\omega ^2\widehat{q}+\nu _1\widehat{q}=0, \end{aligned}$$

where $\widehat{M}=m_v+m_f^{(1)}+m_f^{(2)}$. Hence, using the above form of $\widehat{X}_1$, the vessel equation leads to a second equation linking $\gamma$ and $\widehat{q}$

2.32 $$\begin{aligned} C\widehat{q}-\gamma \frac{2(\rho _1-\rho _2)h_1^0\omega ^2}{\alpha }\sin ^2\left( \frac{1}{2}\alpha L\right) =0, \end{aligned}$$

where

$$\begin{aligned} C=\nu _1-M\omega ^2-\frac{2(\rho _1-\rho _2)\omega ^4}{g\alpha ^3}\sin \left( \frac{1}{2}\alpha L\right) \cos \left( \frac{1}{2}\alpha L\right) +\frac{(\rho _1-\rho _2)\omega ^4L}{g\alpha ^2}. \end{aligned}$$

Solving (2.31) and (2.32) for non-trivial solutions leads to a characteristic equation for the frequency $\omega$ of the form

2.33 $$\begin{aligned} \Delta (s)=P(s)D(s)=0, \end{aligned}$$

where

$$\begin{aligned} P(s)=\sin (s),~~~\hbox {and}~~~D(s)=\cos (s)\left( G-Rs^2-s\tan (s)\right) , \end{aligned}$$

and

$$\begin{aligned} G=\frac{\nu _1 L(\rho _1h_2^0+\rho _2h_1^0)^2}{4(\rho _1-\rho _2)^3(h_1^0h_2^0)^2g},~~R=-1+\frac{\widehat{M}(\rho _1h_2^0+\rho _2h_1^0)}{(\rho _1-\rho _2)^2h_1^0h_2^0L},~~s=\frac{1}{2}\alpha L. \end{aligned}$$

Once the value of *s* is found from (2.33) then $\omega$ is given by

$$\begin{aligned} \omega =\frac{2s}{L}\sqrt{\frac{h_1^0h_2^0g(\rho _1-\rho _2)}{\rho _1h_2^0+\rho _2h_1^0}}. \end{aligned}$$

A full discussion of the properties of this characteristic equation can be found in [13]. Of interest to us in this paper are the linear forms

2.34 $$\begin{aligned} x_1(a,\tau )= & {} a+\left[ \frac{\beta \widehat{q}}{\alpha ^2}\left( \cos (\alpha a)-1\right) +\gamma \sin (\alpha a)\right] \cos (\omega \tau ), \end{aligned}$$

2.35 $$\begin{aligned} \widehat{h}_1(a,\tau )= & {} h_1^0+h_1^0\alpha \left[ \frac{\beta \widehat{q}}{\alpha ^2}\sin (\alpha a)-\gamma \cos (\alpha a)\right] \cos (\omega \tau ), \end{aligned}$$

which we will use to check the validity of the numerical scheme. We are interested in results away from the resonance case (i.e. $D(s)=0$, with $P(s)\ne 0$). In this case, $\sin (s)\ne 0$ in (2.31), and hence, $\gamma =\frac{\beta \widehat{q}}{\alpha ^2}\tan \left( \frac{1}{2}\alpha L\right)$.

## Variational discretization and computation

### Numerical algorithm

To formulate the numerical scheme, we discretize the Lagrangian state space into *N* parcels by setting

3.1 $$\begin{aligned} a_i=(i-1)\Delta a,~~~i=1,\ldots ,N+1,~~~\hbox {with}~~~\Delta a=\frac{L}{N}. \end{aligned}$$

Let $x_i(t):=x_1(a_i,\tau )$ (note the dropping of the subscript ‘1’ on the *x* here) and $w_i(t):=w(a_i,\tau )$. The derivatives are discretized using forward differences, except when $i=N+1$ where backward differences are used, and the integrals are approximated using the trapezoidal rule.

Equations (2.24)–(2.26) can be discretized in a straightforward manner, as the variables for which variations are taken, do not appear differentiated with respect to *a* on the RHS of the equations. However, in (2.23), derivatives of $x_{1}$ with respect to *a* do appear in the RHS, and thus, it is not clear how to discretize these equations. To overcome this, we use a semi-discretization of the Hamiltonian, where the Hamiltonian is discretized and then variations with respect to $x_i$ are taken.

To form the semi-discretization, we note that the discretized form of $B(a,\tau )$ is $B_i=B(a_i,\tau )$ such that

$$\begin{aligned} B_i= & {} \frac{\chi _i\left( d(x_{i+1}-x_i)-\Delta a\chi _i\right) (\rho _1-\rho _2)}{\Gamma _i},~~i=1,...,N,\\ \Gamma _i= & {} \rho _1\left( d(x_{i+1}-x_i)-\Delta a\chi _i\right) +\rho _2\Delta a\chi _i,~~i=1,...,N,\\ B_{N+1}= & {} \frac{\chi _{N+1}\left( d(x_{N+1}-x_N)-\Delta a\chi _{N+1}\right) (\rho _1-\rho _2)}{\Gamma _{N+1}},\\ \Gamma _{N+1}= & {} \rho _1\left( d(x_{N+1}-x_N)-\Delta a\chi _{N+1}\right) +\rho _2\Delta a\chi _{N+1}. \end{aligned}$$

Therefore the integrals which appear in $\mathscr {H}$ can be approximated using the trapezoidal rule

$$\begin{aligned} F_1(x_1,...,x_{N+1})=\int _0^LB\lambda \,\mathrm{d}a\approx (\rho _1-\rho _2)\sum _{i=1}^N\left[ \frac{\chi _i\left( d(x_{i+1}-x_i)-\Delta a\chi _i\right) }{\Gamma _i}\right] \lambda _i\Delta a, \end{aligned}$$

where $\lambda$ denotes either $w^2$, *w* or 1, and therefore it can be shown that

3.2 $$\begin{aligned} \frac{\delta F_1}{\delta x_i}\approx (\rho _1-\rho _2)\rho _2d\Delta a^2\left[ -\frac{\chi _i^2\lambda _i}{\Gamma _i^2}+\frac{\chi _{i-1}^2\lambda _{i-1}}{\Gamma _{i-1}^2}\right] ,~~~\hbox {for}~~~i=2,...,N. \end{aligned}$$

We also need to take variations with respect to $x_i$ which occur in $A^{-1}$, and again it is simple to show that

$$\begin{aligned} \frac{\delta A^{-1}}{\delta x_i}\approx \frac{(\rho _1-\rho _2)^2\rho _2d\Delta a^2}{A^2}\left[ -\frac{\chi _i^2}{\Gamma _i^2}+\frac{\chi _{i-1}^2}{\Gamma _{i-1}^2}\right] ,~~~\hbox {for}~~~i=2,...,N. \end{aligned}$$

Finally from [1] we note that

$$\begin{aligned} \frac{\delta F_2}{\delta x_1}\approx \Delta a^2\left[ \frac{\chi _j^2}{(x_{j+1}-x_j)^2}-\frac{\chi _{j-1}^2}{(x_{j}-x_{j-1})^2}\right] , \end{aligned}$$

where

$$\begin{aligned} F_2(x_1,...,x_{N+1})=\int _0^L\frac{\chi ^2}{x_{1a}}\,\mathrm{d}a\approx \Delta a^2\sum _{i=1}^N\frac{\chi _i^2}{(x_{i+1}-x_i)}. \end{aligned}$$

Hence, the full discretized form of Hamilton’s equations to leading order are

3.3 $$\begin{aligned} (w_i)_\tau= & {} \frac{d\rho _2\Delta a}{2\chi _i}\left[ \frac{\chi _i^2w_i^2}{\Gamma _i^2}-\frac{\chi _{i-1}^2w_{i-1}^2}{\Gamma _{i-1}^2}\right] -\frac{(p-I)(\rho _1-\rho _2)\rho _2d\Delta a}{A\chi _i}\left[ \frac{\chi _i^2w_i}{\Gamma _i^2}-\frac{\chi _{i-1}^2w_{i-1}}{\Gamma _{i-1}^2}\right] \nonumber \\&+\,\frac{(p-I)^2(\rho _1-\rho _2)^2\rho _2d\Delta a}{2A^2\chi _i}\left[ \frac{\chi _i^2}{\Gamma _i^2}-\frac{\chi _{i-1}^2}{\Gamma _{i-1}^2}\right] \nonumber \\&-\,\frac{g(\rho _1-\rho _2)\Delta a}{2\chi _i}\left[ \frac{\chi _i^2}{(x_{i+1}-x_i)^2}-\frac{\chi _{i-1}^2}{(x_{i}-x_{i-1})^2}\right] ,~~~i=2,\ldots ,N-1, \end{aligned}$$

3.4 $$\begin{aligned} p_\tau= & {} -\nu _1q+\nu _2q^3, \end{aligned}$$

3.5 $$\begin{aligned} (x_i)_\tau= & {} \frac{(d(x_{i+1}-x_i)-\chi _i\Delta a)}{\Gamma _i}w_i\nonumber \\&-\,\frac{(d(x_{i+1}-x_i)-\chi _i\Delta a)}{\Gamma _i}\frac{(\rho _1-\rho _2)}{A}(p-I),~~i=2,\ldots ,N-1, \end{aligned}$$

3.6 $$\begin{aligned} q_\tau= & {} \frac{(p-I)}{A}. \end{aligned}$$

This gives a set of 2*N* equations for the $2N+4$ unknowns. The remaining 4 equations come from the boundary conditions

$$\begin{aligned}&x_1=0~~~\hbox {and}~~~x_{N+1}=L,\\&w_1=w_{N+1}=(\rho _1-\rho _2)A^{-1}(p-I). \end{aligned}$$

The discretized set of equations can be written as

3.7 $$\begin{aligned} \mathbf{p}_\tau =f(\mathbf{p},\mathbf{q}),~~~\mathbf{q}_\tau =g(\mathbf{p},\mathbf{q}), \end{aligned}$$

where we define $\mathbf{p}=(p,w_1,\ldots ,w_{N+1})$ and $\mathbf{q}=(q,x_1,\ldots ,x_{N+1})$. This form of the equations is amenable to time integration by a geometric integration scheme, namely the *implicit-midpoint rule* approach. In this case, the system becomes the set of nonlinear algebraic equations

$$\begin{aligned} \mathbf{p}^{n+1}= & {} \mathbf{p}^n+\Delta \tau f\left( \frac{\mathbf{p}^n+\mathbf{p}^{n+1}}{2},\frac{\mathbf{q}^n+\mathbf{q}^{n+1}}{2}\right) {,}\\ \mathbf{q}^{n+1}= & {} \mathbf{q}^n+\Delta \tau g\left( \frac{\mathbf{p}^n+\mathbf{p}^{n+1}}{2},\frac{\mathbf{q}^n+\mathbf{q}^{n+1}}{2}\right) {,} \end{aligned}$$

where *n* denotes the time-step, such that $\mathbf{p}^n=\mathbf{p}(n\Delta \tau )$. This system of implicit equations are solved at each new time step via Newton iterations. In order to increase the speed of the iteration scheme, the method of [18] is employed to iteratively calculate the inverse Jacobian matrix after the first iteration of the first time step.

In this paper, we consider the following initial condition from the linear solution which is derived in Sect. 2.2

$$\begin{aligned} q(0)=\widehat{q},~~q_\tau (0)=0,~~x(a,0)=a+\widehat{q}_1\widetilde{X}_1(a),~~\widehat{h}(a,0)=h_{1}^{0}-\widehat{q}_2h_{1}^0\frac{\mathrm{d}\widetilde{X}_1}{\mathrm{d}a}(a),~~w(a,0)=0,~~p(0)=0, \end{aligned}$$

where

$$\begin{aligned} \widetilde{X}_1(a)=\frac{\widehat{X}_1}{\widehat{q}}=\frac{\beta }{\alpha ^2}\left( \cos (\alpha a)-1+\tan \left( \frac{1}{2}\alpha L\right) \sin (\alpha a)\right) , \end{aligned}$$

from (2.30). The value of $\widehat{q}$ is the initial displacement of the vessel from its equilibrium point, while $\widehat{q}_1$ and $\widehat{q}_2$ are chosen as independent parameters. When $\widehat{q}_1=\widehat{q}_2=\widehat{q}$ the initial condition gives the linear solution derived in Sect. 2.2 and we can verify our results against the exact solution, when $\widehat{q}_1=0$ with $\widehat{q}_2$ and $\widehat{q}$ independent, then we have the same initial condition as in [13], and thus, we can verify against their nonlinear results, and finally when $\widehat{q}_1=\widehat{q}_2=0$, we have an initial condition akin to those achievable in an experiment, namely a horizontally displaced vessel released from rest with a quiescent fluid.

### Numerical results

In this section, we present results of the numerical scheme for several sets of parameter values. In order to validate the numerical scheme, we compare our results both with the linear solution, and the nonlinear f-wave numerical scheme results presented in [13]. Once validated we then present results in the non-Boussinesq limit, a limit which the f-wave scheme struggles to resolve due to difficulties satisfying the system constraints. For the results presented we set $N=200$ and $\Delta \tau =10^{-3}$ (although $N=50$ and $\Delta \tau =10^{-2}$ are sufficient for the linear results).

**Table 1:** Simulation parameter values for Figs. 2, 3, 4, 5, 6, 7, 8, 9, 11 and 12

Parameter (units)	Figs. 2, 3	Figs. 4, 5	Figs. 6, 7	Figs. 8, 9	Figs. 11, 12
*L* (m)	1	1	1	1	1
*d* (m)	0.12	0.12	0.08	0.12	0.12
$h_1^0$ (m)	0.06	0.06	0.04	0.08	0.04
$h_2^0$ (m)	0.06	0.06	0.04	0.04	0.04
$h_3^0$ (m)	–	–	–	–	0.04
$\rho _1$ ($\mathrm{kg\,m}^{-3}$)	1000	1000	1000	1000	1000
$\rho _2$ ($\mathrm{kg\,m}^{-3}$)	900	1	700	1	500
$\rho _3$ ($\mathrm{kg\,m}^{-3}$)	–	–	–	–	1
$m_f^{(1)}$ (kg)	60	60	40	80	40
$m_f^{(2)}$ (kg)	54	0.06	28	0.04	20
$m_f^{(3)}$ (kg)	–	–	–	–	0.04
$m_v$ (kg)	10	10	3.4	10	10
$\widehat{q}$ (m)	$1\times 10^{-4}$	$1\times 10^{-4}$	0.01	0.07	0.07
$\widehat{q}_1$ (m)	$1\times 10^{-4}$	$1\times 10^{-4}$	0	0	–
$\widehat{q}_2$ (m)	$1\times 10^{-4}$	$1\times 10^{-4}$	0.1	0	–
$\nu _1$ ($\mathrm{kg\,s}^{-2}$)	100	100	189.40	100	100
$\nu _2$ ($\mathrm{kg\,m}^{-2}\mathrm{s}^{-2}$)	0	0	–189.40	800	800
$\omega$ ($\mathrm{s^{-1}}$)	0.8995	1.0980	–	–	–

Results are presented for the vessel evolution *q*(*t*) and the surface interface evolution $h_1(x_1,t)$ along with time evolutions of the total vessel energy $E_v(t)$ and the total fluid energy $E_f(t)$ defined by

$$\begin{aligned} E_v= & {} \frac{m_v}{2A^2}\left( p-I\right) ^2+\frac{1}{2}\nu _1q^2-\frac{1}{4}\nu _2q^4,\\ E_f= & {} \frac{1}{2(\rho _1-\rho _2)}\int _0^LBw^2\,\mathrm{d}a+\frac{\rho _1\rho _2d^2}{2A^2}\left( p-I\right) ^2\int _0^L\frac{x_{1a}^2}{\Gamma }\,\mathrm{d}a+\frac{g}{2}(\rho _1-\rho _2)\int _0^L\frac{\chi ^2}{x_{1a}}\,\mathrm{d}a+\frac{1}{2}gd^2\rho _2L. \end{aligned}$$

It is also possible to show via simple algebraic manipulation that

$$\begin{aligned} \rho _1\rho _2d^2\int _0^L\frac{x_{1a}^2}{\Gamma }\,\mathrm{d}a=m_f^{(1)}+m_f^{(2)}-(\rho _1-\rho _2)\int _0^LB\,\mathrm{d}a=A-m_v, \end{aligned}$$

and thus $E_v+E_f=\mathscr {H}$. Therefore the Hamiltonian is the total energy of the system, and thus, the discretized form of $\mathscr {H}$

3.8 $$\begin{aligned} \mathscr {H}_N= & {} \frac{1}{2(\rho _1-\rho _2)}\Delta a\sum _{j=1}^NB_jw_j^2+\frac{1}{2A_N}\left( p-I_N\right) ^2\nonumber \\&+\,\frac{1}{2}(\rho _1-\rho _2)g\Delta a^2\sum _{j=1}^N\frac{\chi _j^2}{(x_{j+1}-x_j)}+\frac{1}{2}\rho _2gd^2L+\frac{1}{2}\nu _1q^2-\frac{1}{4}\nu _2q^4, \end{aligned}$$

is conserved along orbits of the semi-discretization (3.3)–(3.6), where

$$\begin{aligned} I_N=\Delta a\sum _{j=1}^NB_jw_j,~~\hbox {and}~~A_N=m_v+m_f^{(1)}+m_f^{(2)}-(\rho _1-\rho _2)\Delta a\sum _{j=1}^NB_j. \end{aligned}$$

The parameter values for the simulations presented in this section are given in Table 1.

**Fig. 2:**
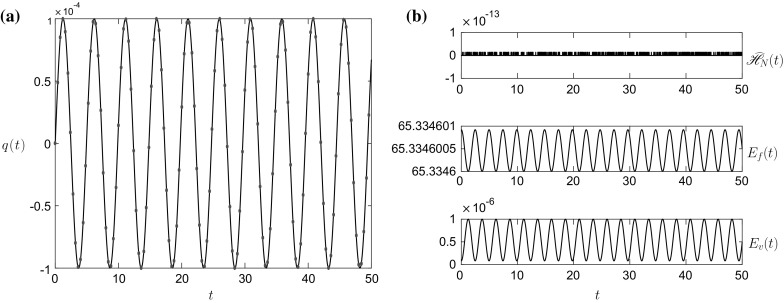
**a** The vessel displacement *q*(*t*) and **b** $E_f(t)$, $E_v(t)$ and $\widehat{\mathscr {H}}_N(t)$ for the linear initial condition $\widehat{q}_1=\widehat{q}_2=\widehat{q}$ and the initial parameter values given in column 1 of Table 1. The *dots* in panel **a** represent the linear solution (2.28). The value of $\omega =0.8995$

**Fig. 3:**
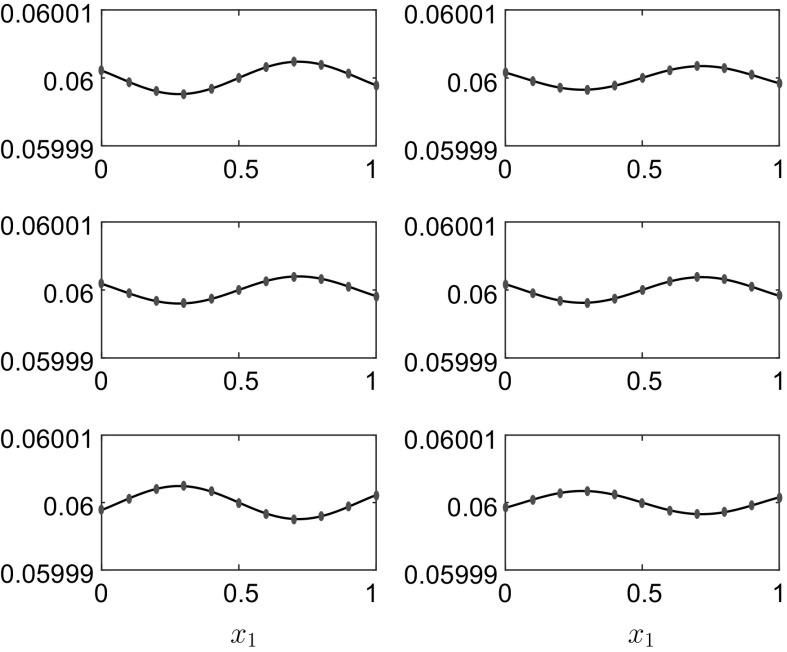
The interface position $h_1(x_1,t)$ for the results in Fig. 2 at, *top row* $t=4$ and $t=8$, *middle row* $t=18$ and $t=29$, *bottom row* $t=41$ and $t=50$. The *dots* represent the linear solution (2.35). The rigid lid is at $h_1^0+h_2^0=0.12\,$m

**Fig. 4:**
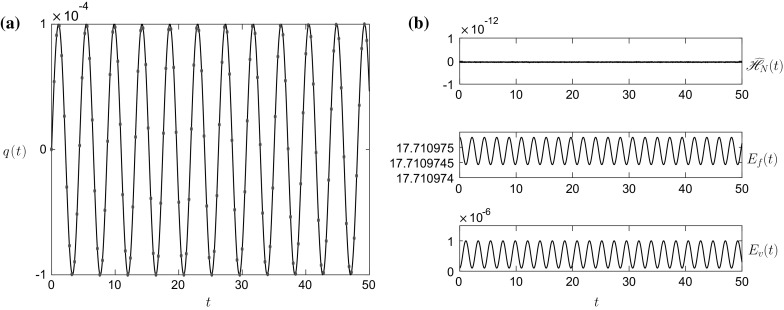
**a** The vessel displacement *q*(*t*) and **b** $E_f(t)$, $E_v(t)$ and $\widehat{\mathscr {H}}_N(t)$ for the linear initial condition $\widehat{q}_1=\widehat{q}_2=\widehat{q}$ and the initial parameter values given in column 2 of Table 1. The *dots* in panel **a** represent the linear solution (2.28). The value of $\omega =1.0980$

**Fig. 5:**
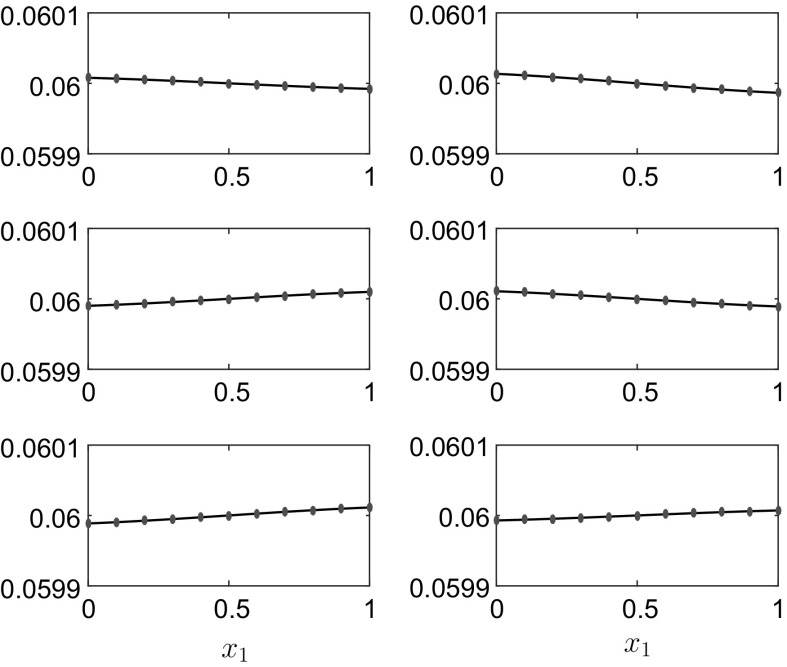
The interface position $h_1(x_1,t)$ for the results in Fig. 4 at, *top row* $t=4$ and $t=8$, *middle row* $t=18$ and $t=29$, *bottom row* $t=41$ and $t=50$. The *dots* represent the linear solution (2.35). The rigid lid is at $h_1^0+h_2^0=0.12\,$m

The linear results in Figs. 2, 3, 4 and 5 show excellent agreement with the exact solution (red dots) in both the Boussinesq (Figs. 2, 3) and non-Boussinesq (Figs. 4, 5) regimes. In both cases, the value of $\omega$ is the 1st (lowest frequency) root of the characteristic equation (2.33). The accuracy of the numerical scheme can be determined by examining the energy error $\widehat{\mathscr {H}}_N(t)=\mathscr {H}_N(t)-\mathscr {H}_N(0)$, given by the top panel of Figs. 2b and 4b. In these linear simulations, the energy conservation is excellent with the error of $O(10^{-14})$.

**Fig. 6:**
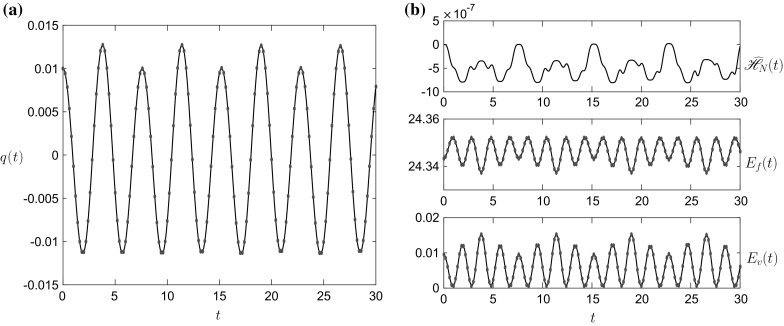
**a** The vessel displacement *q*(*t*) and **b** $E_f(t)$, $E_v(t)$ and $\widehat{\mathscr {H}}_N(t)$ for the initial condition $\widehat{q}_1=0,~\widehat{q}_2\ne \widehat{q}$ and the initial parameter values given in column 3 of Table 1. The *dots* in panel **a** represent the f-wave solution from [13]

**Fig. 7:**
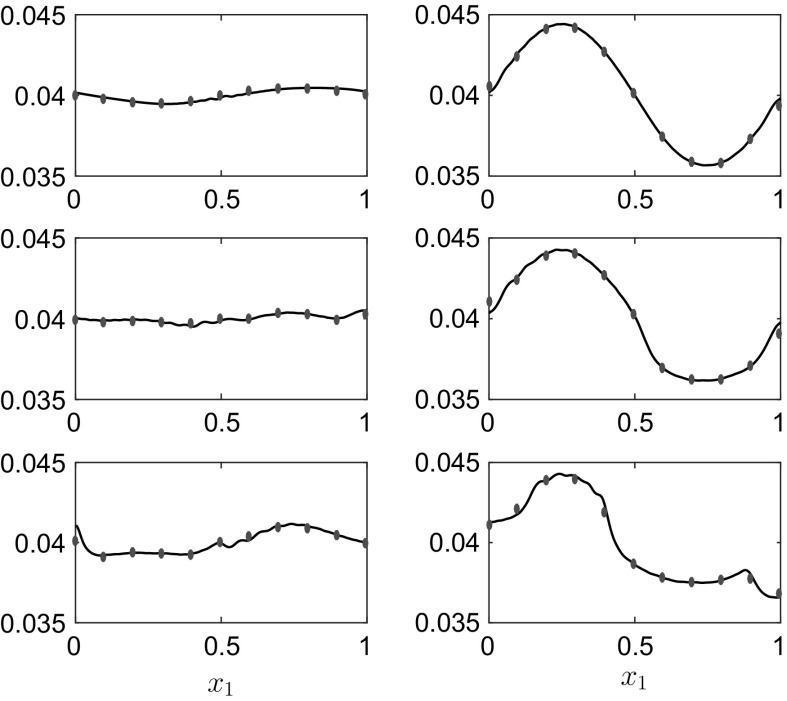
The interface position $h_1(x_1,t)$ for the results in Fig. 6 at, *top row* $t=1.9$ and $t=7.6$, *middle row* $t=17.1$ and $t=22.8$, *bottom row* $t=28.5$ and $t=30$. The *dots* represent the f-wave solution from [13]. The rigid lid is at $h_1^0+h_2^0=0.08\,$m

Figures 2, 3, 4 and 5 validate the numerical LPP approach in the linear regime, but it can also be validated it in the nonlinear regime by comparing against the f-wave simulations of [13] in Figs. 6 and 7. The parameter values for this simulation are given in column 3 of Table 1.

The dots in both figures signify the results of [13], and the agreement is excellent. There are some minor discrepancies in the fluid interface profiles in Fig. 7, mainly close to the side walls, but these differences do not manifest themselves into the vessel evolution on the simulation time-scale. The energy error $\widehat{\mathscr {H}}_N(t)$, in Fig. 6b is again small, $O(10^{-6})$, and bounded.

**Fig. 8:**
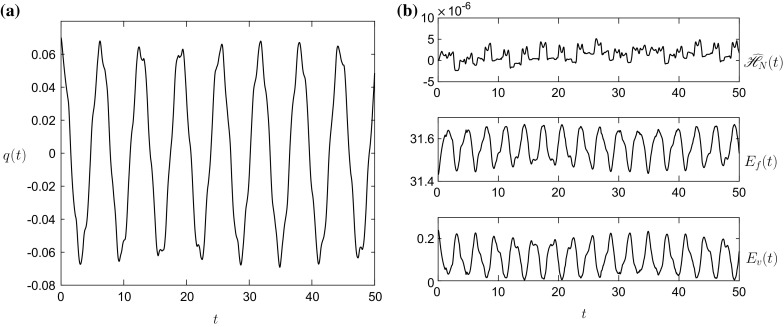
**a** The vessel displacement *q*(*t*) and **b** $E_f(t)$, $E_v(t)$ and $\widehat{\mathscr {H}}_N(t)$ for the initial condition $\widehat{q}_1=\widehat{q}_2=0,~\widehat{q}\ne 0$ and the initial parameter values given by column 4 of Table 1

**Fig. 9:**
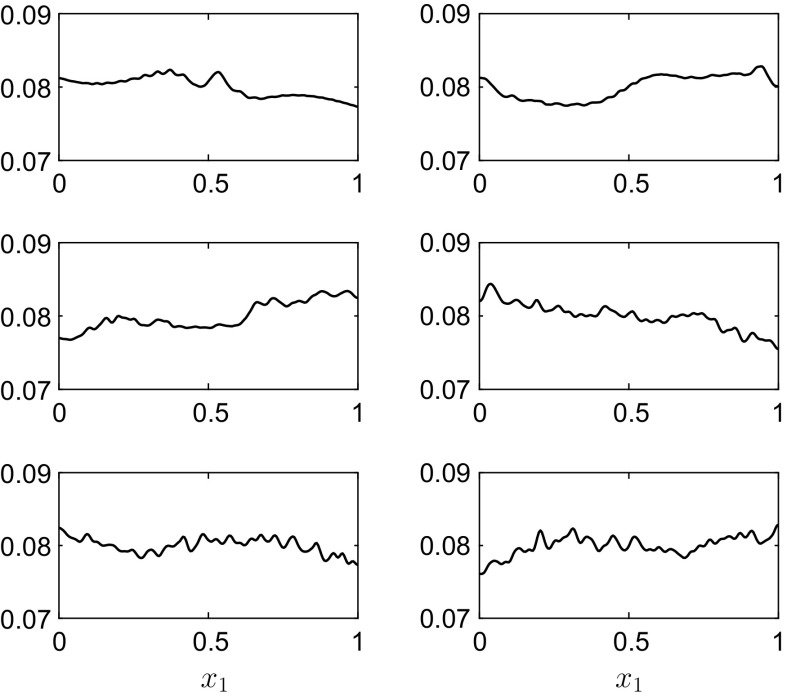
The interface position $h_1(x_1,t)$ for the results in Fig. 8 at, *top row* $t=4$ and $t=8$, *middle row* $t=18$ and $t=29$, *bottom row* $t=41$ and $t=50$. The rigid lid is at $h_1^0+h_2^0=0.12\,$m

The density ratio $\rho _2/\rho _1=0.7$ in Figs. 6 and 7 is on the borderline between the Boussinesq and non-Boussinesq regimes. The f-wave numerical scheme developed by [13] works most effectively in the Boussinesq regime, especially for weakly nonlinear simulations. The scheme encounters problems satisfying the system constraints for density ratios $\rho _1/\rho _1\lesssim 0.7$. The Hamiltonian scheme developed here has the rigid lid and mass-flux conditions (2.1) and (2.16) directly built in to the scheme and so is able to resolve simulations for for these density ratios. Figs. 8 and 9 show results for $\rho _2/\rho _1=10^{-3}$, which is the density ratio of air to water for an initial condition akin to those found in an experimental setup, $\widehat{q}_1=\widehat{q}_2=0$. As the initial interface is flat, the initial condition consists of an infinite sum of all the sloshing modes in (2.33) at different amplitudes, and thus, the result is the lowest frequency mode superposed with higher frequency modes, giving the small oscillations on the results. The energy error $\widehat{\mathscr {H}}_N(t)$ in Fig. 8b, although larger than the result in Fig. 6b, is still relatively small $O(10^{-5})$, and bounded for the time-scale of the simulations. The results in Fig. 9 depict the interface gently sloshing back and forth in the vessel, and as it does so it becomes increasingly more fine scaled. This is a well known characteristic when symplectic schemes are applied to sloshing problems [19] and is due to the energy of the system cascading down to the high frequency modes, in what is essentially a spectral scheme. However, as the numerical time integrator is symplectic, it conserves this energy and so this energy remains in the high frequency modes as these high frequency oscillations. These could be removed using an artificial viscosity term or the filtering scheme used by [20], but the numerical scheme will then no longer be energy conserving.

The two-layer results presented here show the capabilities of the Hamiltonian approach for these multilayer sloshing problems. Note, however, despite the introduction of the mapping $\phi (a_1,\tau )$ to ensure $x_1(a_1,\tau )=x_2(\phi (a_1,\tau ),\tau )$, this mapping was never discussed or plotted. This is because the two-layer problem is in fact a special case of the multilayer sloshing problem, because equations (2.1) and (2.16) mean that the upper-layer variables can be eliminated and the problem can be formulated solely in terms of lower-layer variables. In the next section, we formulate the general *M*-layer shallow-water problem, and present results for three-layer sloshing, where the mappings $\phi _i$ do need to be calculated.

## Extension to multilayer shallow-water flows

**Fig. 10:**
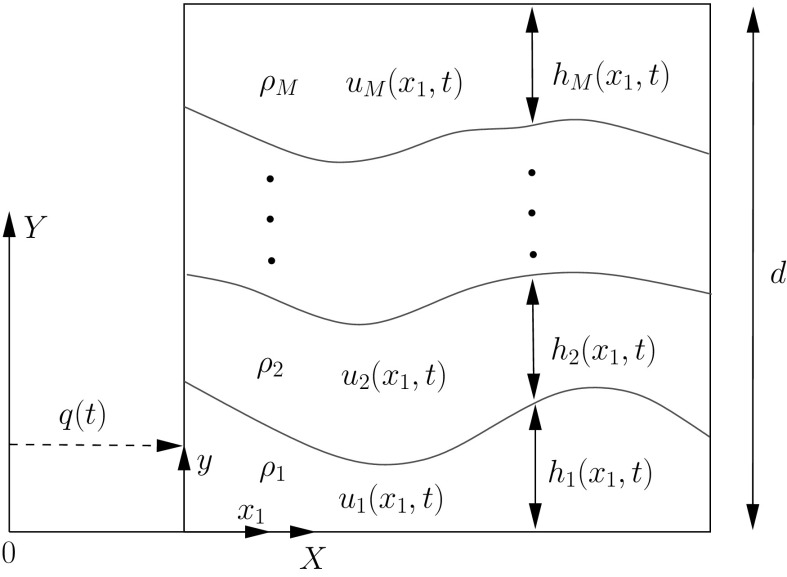
Schematic of *M*-layer shallow-water sloshing in a moving rectangular vessel, with the constraint $x_1=x_2=\cdots =x_M$ imposed

The extension of the theory to the *M*-layer shallow-water problem is straightforward, with the biggest difference being the necessity to calculate the mapping functions $\phi _i(a_1,\tau )$. The derivation and analysis can get a bit lengthy so detail is recorded in Appendix 3 for the three-layer case. A schematic of the general *M*-layer problem is shown in Fig. 10. Here we will impose the constraint $x_1=x_2=...=x_M$ from the outset in order to simplify the analysis.

The $i\mathrm{th}$ layer mass conservation and momentum equations (for $i=1, ...,M$) in the *M*-layer shallow-water equations with a rigid lid are

4.1 $$\begin{aligned}&\frac{\partial }{\partial t}\left( \rho _ih_i\right) +\frac{\partial }{\partial x_1}\left( \rho _ih_iu_i\right) =0, \end{aligned}$$

4.2 $$\begin{aligned}&\frac{\partial }{\partial t}\left( \rho _iu_i\right) +\frac{\partial }{\partial x_1}\left( \frac{1}{2}\rho _iu_i^2+g\left[ \sum _{j=i+1}^M\rho _jh_j+\rho _i\sum _{j=1}^ih_j\right] +p_s\right) =-\rho _i\ddot{q}, \end{aligned}$$

with the Eulerian rigid lid constraint

4.3 $$\begin{aligned} \sum _{j=1}^Mh_j=d. \end{aligned}$$

The derivation of this multilayer system is given in Appendix 2. The associated generalised vessel equation to (2.6) is

4.4 $$\begin{aligned} \frac{\mathrm{d}}{\mathrm{d}t}\left[ \int _0^L\sum _{i=1}^M\rho _ih_iu_i\,\mathrm{d}x_1+\left( m_v+\sum _{i=1}^Mm_f^{(i)}\right) \dot{q}\right] +\nu _1q-\nu _2q^3=0. \end{aligned}$$

This multilayer shallow-water system is the Euler–Lagrange equation associated with the following Lagrangian functional in the Eulerian setting

4.5 $$\begin{aligned} \mathscr {L}(h_1,...,h_M,u_1,...,u_M,q,\dot{q})=\int _{t_1}^{t_2}\int _0^L \widehat{L}\,\mathrm{d}x_1\mathrm{d}t+\int _{t_1}^{t_2}\left( \frac{1}{2}m_v\dot{q}^2-\frac{1}{2}\nu _1q^2+\frac{1}{4}\nu _2q^4\right) \,\mathrm{d}t, \end{aligned}$$

where

$$\begin{aligned} \widehat{L}= & {} \sum _{j=1}^M L_j - g\sum _{j=1}^{M-1}h_j\left[ \sum _{k=j+1}^M\rho _kh_k\right] -p_s\left( \sum _{j=1}^Mh_j-d\right) ,\\ L_j= & {} \frac{1}{2}\rho _jh_j\left( u_j+\dot{q}\right) ^2-\frac{1}{2}\rho _jgh_j^2+\rho _jf_j\left( h_{jt}+(h_ju_j)_{x_1}\right) ,\\ \end{aligned}$$

and $p_s$ and $f_j$ are Lagrange multipliers. In order to construct a geometric integration scheme such as that used in Sect. 2, we must first transform the equations from the Eulerian to the Lagrangian description, secondly construct a Lagrangian functional in the LPP description, and then Legendre transform to obtain the Hamiltonian form. To do this, we first note that as in Sect. 2 we have two additional equations, the constraint (4.3), and the corresponding mass flux conservation equation

4.6 $$\begin{aligned} \sum _{j=1}^Mh_ju_j=0, \end{aligned}$$

which can be derived in the same way as for the two-layer case. As in the two-layer system, these two equations are used to eliminate $u_i$ and $h_i$ for one layer, which w.l.o.g, we choose to be the upper layer, with suffix *M*. Now introducing the LPP mapping (2.8) into the layer 1 mass conservation equation leads again to (2.11) and hence (2.15). Thus, $\widehat{h}_1$ is now written in terms of $x_1$ only, with $u_1=x_{1\tau }$ its associated momenta. However, unlike the two-layer case, we still have layer variables $(h_2,u_2),\ldots ,(h_{M-1},u_{M-1})$ to eliminate from the Lagrangian and replace by some position variable and its associated momenta.

If we now consider the constraint that

$$\begin{aligned} x_1(a,\tau )=x_i(\phi _i(a,\tau ),\tau ),~~~i=2,\ldots ,M-1, \end{aligned}$$

where $\phi _i(a,\tau )$ is a mapping variable, then we can show that in each layer the mass conservation equation in the LPP framework reduces to

$$\begin{aligned} \frac{\partial }{\partial \tau }\left( \widehat{h}_ix_{i\phi _i}\right) =0,~~~\Longrightarrow ~~~\widehat{h}_i=\frac{\chi _i(\phi _i)}{x_{i\phi _i}},~~~i=2,\ldots ,M-1. \end{aligned}$$

Now by noting that $x_{1a}=x_{i\phi _i}\phi _{ia}$, then

4.7 $$\begin{aligned} \widehat{h}_i=\frac{\chi _i(\phi _i)\phi _{ia}}{x_{1a}},~~~i=2,\ldots ,M-1. \end{aligned}$$

Similarly

4.8 $$\begin{aligned} u_i=\left. \frac{\partial x_i}{\partial \tau }\right| _{\phi ~\mathrm{fixed}}=x_{1\tau }-x_{i\phi _i}\phi _{i\tau }=x_{1\tau }-\frac{x_{1a}\phi _{i\tau }}{\phi _{ia}}. \end{aligned}$$

Thus, using (4.3), (4.6), (2.15), (4.7) and (4.8) the multilayer Lagrangian

$$\begin{aligned} \mathscr {L}=\mathscr {L}(h_1,\ldots ,h_M,u_1,\ldots ,u_M,q,\dot{q}), \end{aligned}$$

in the Eulerian framework becomes

$$\begin{aligned} \mathscr {L}=\mathscr {L}(x_1,\phi _2,\ldots ,\phi _{M-1},x_{1\tau },\phi _{2\tau },\ldots ,\phi _{(M-1)\tau },q,q_\tau ), \end{aligned}$$

in the LPP framework, i.e. it is written in terms of position variables and their associated momenta. Therefore using the Legendre transformation a Hamiltonian

$$\begin{aligned} \mathscr {H}=\mathscr {H}(x_1,\phi _2,\ldots ,\phi _{M-1},x_{1\tau },\phi _{2\tau },\ldots ,\phi _{(M-1)\tau },q,q_\tau ), \end{aligned}$$

can be constructed, and the geometric integration scheme of Sect. 3 applied to it.

**Fig. 11:**
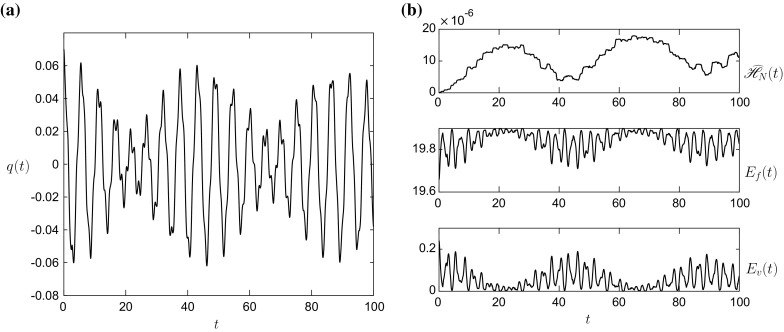
**a** The vessel displacement *q*(*t*) and **b** $E_f(t)$, $E_v(t)$ and $\widehat{\mathscr {H}}_N(t)$ for the initial condition (4.9) and the initial parameter values given in column 5 of Table 1

**Fig. 12:**
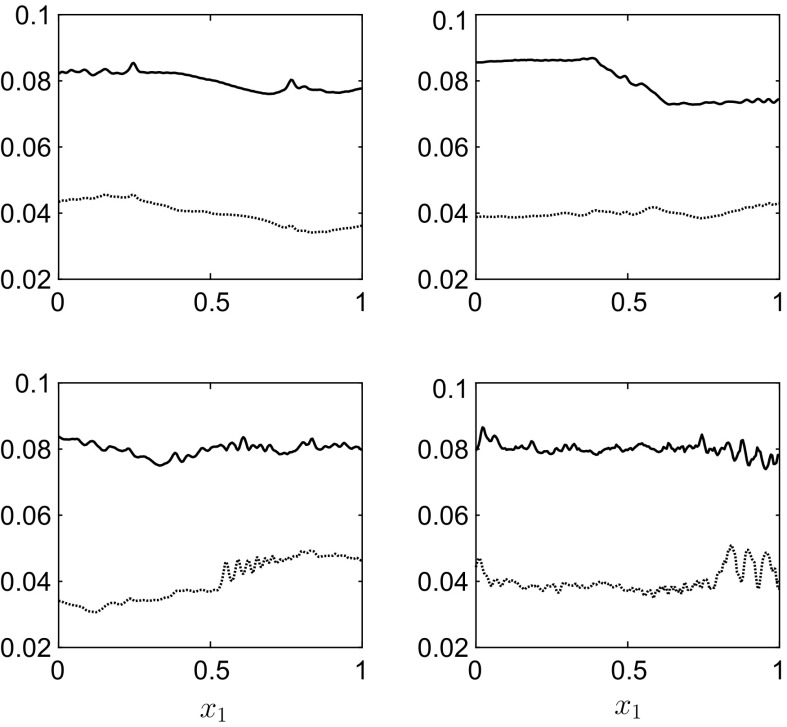
The interface positions $h_1(x_1,t)$ (*lower curves* in each panel) and $h_1(x_1,t)+h_2(x_1,t)$ (*upper curves* in each panel) for the results in Fig. 11 at, *top row* $t=4$ and $t=8$, *bottom row* $t=18$ and $t=29$. The rigid lid is at $h_1^0+h_2^0+h_3^0=0.12\,$m

### Numerical implementation for three layers

To demonstrate that the numerical scheme of Sect. 3 generalises to the *M*-layer problem, we present a result for coupled three-layer sloshing in Figs. 11 and 12. Details of the derivation of the three-layer Hamiltonian and symplectic integration scheme, as well as validation of the scheme, are given in Appendix 3. The initial conditions for these simulations are

4.9 $$\begin{aligned} q(0)=\widehat{q},~~q_\tau (0)=0,~~x_1(a,0)=a,~~\phi _2(a,0)=a,~~\widehat{h}_1(a,0)=h_1^0,~~\widehat{h}_2(a,0)=h_2^0, \end{aligned}$$

with the simulation parameter values given in column 5 of Table 1. In this simulation, we use $N=200$ and $\Delta \tau =10^{-4}$.

The results in Figs. 11 and 12 can be directly compared with those in Figs. 8 and 9, as they are essentially the same parameter values except for the inclusion of a third, less-dense, middle layer. These results show a vessel motion whose amplitude is strongly modulated by the inclusion of the third layer. This modulated vessel displacement is due to the hydrodynamic force on the vessel walls slowly becoming out of phase with the restoring force of the spring, before slowly moving back in phase. This more complex behaviour is not a surprise as the characteristic equation for this system (8.40) has more solutions compared to the two-layer equation (2.33) due to the inclusion of the additional interface. The interface profiles again show fine scale structure at later times, but at $t=29$ there exists fairly large oscillations at the lower interface. Also, the energy error $\widehat{\mathscr {H}}_N(t)$ in Fig. 11b, while still small, $O(10^{-5})$, grows moderately over the time frame of the simulation. The reason for these two observations, we believe, is due to the Kelvin–Helmholtz instability on the interface [21], and we use a smaller time-step to stop the error growing more rapidly. This is more evident in the validation simulation in Appendix 3. Hence, one has to check the energy error $\widehat{\mathscr {H}}_N(t)$ for a calculation to determine whether it is still within tolerable bounds. Again the introduction of artificial viscosity or filtering would help limit this instability by removing the fine-scale high-frequency modes from the system, which grow fastest in an inviscid system [22].

## Conclusions and discussion

This paper documents the Lagrangian variational formulation of the LPP representation of multilayer shallow-water sloshing, coupled to horizontal vessel motion governed by a nonlinear spring. The Lagrangian variational formulation was transformed to a Hamiltonian formulation which has nice properties for numerical simulation. A symplectic numerical integration scheme was applied to the resulting set of Hamiltonian partial differential equations for the two-layer problem, and results of the simulations were found to be in excellent agreement with the linear solution and the nonlinear results of the f-wave scheme of [13]. Using this Hamiltonian formulation the results of [13] were extended into the non-Boussinesq regime, with a result presented for a density ratio $\rho _2/\rho _1=10^{-3}$, akin to that of air over water.

The Hamiltonian formulation was presented in detail for the two-fluid system, but the solution procedure was generalised in Sect. 4 to a system of *M*-fluid layers coupled to horizontal vessel motion where the vessel is attached to a nonlinear spring. Results were presented for a three-layer system, with the full derivation confined to Appendix 3. Results for the three-layer system showed a system energy error which grew slowly in time, due to the Kelvin-Helmholtz instability on the fluid interfaces. For the results presented in this paper, this error growth was small and thus tolerable for the time frame of the simulations. However, this error would need to be examined in fully nonlinear simulations or long-time simulations to make sure it was small compared to the fluid velocities and vessel displacement. Also, in thin layers, where fluid velocities tend to be larger to conserve the mass flux (4.6), this instability could be an issue. Surface tension or a filter could be added to mollify the instability.

As this work was motivated by studying the WEC of Offshore Wave Energy Ltd (OWEL), a direction of great interest is to extend the vessel motion to incorporate rotation (pitch) along with the translations considered here, and to incorporate influx-efflux boundary conditions at the side walls, which model the waves entering the device and leaving through the extraction route. In the OWEL WEC, the wetting and drying of the upper rigid lid is very important for the modelling of the power-take-off mechanism. The current two-layer approach considered in this paper cannot incorporate this phenomena. The reason for this comes from the mass-flux equation $h_1u_1+h_2u_2=0$ which holds throughout the fluid. We find that as $h_2\rightarrow 0$ in this expression, despite the momentum $h_2u_2$ reducing in size, the value of $u_2$ becomes large which causes numerical difficulties in the current scheme. Thus, another area of great interest is to incorporate this feature into the model.

## Figures and Tables

**Fig. 13:**
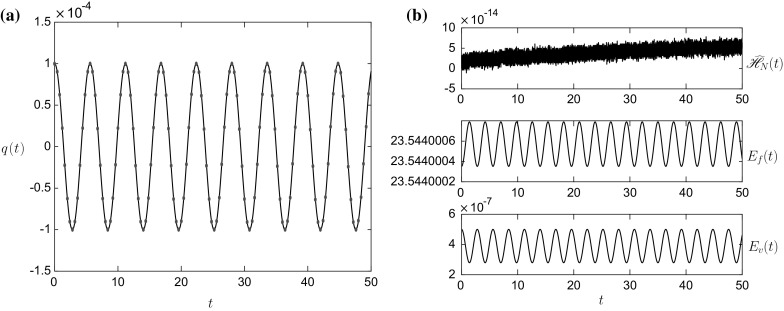
**a** The vessel displacement *q*(*t*) and **b** $E_f(t)$, $E_v(t)$ and $\widehat{\mathscr {H}}_N(t)$ for the linear initial condition $Q_1=1$ and the initial parameter values given in column 1 of Table 2. The *dots* represent the linear solution (8.26) for the lowest-frequency mode

**Fig. 14:**
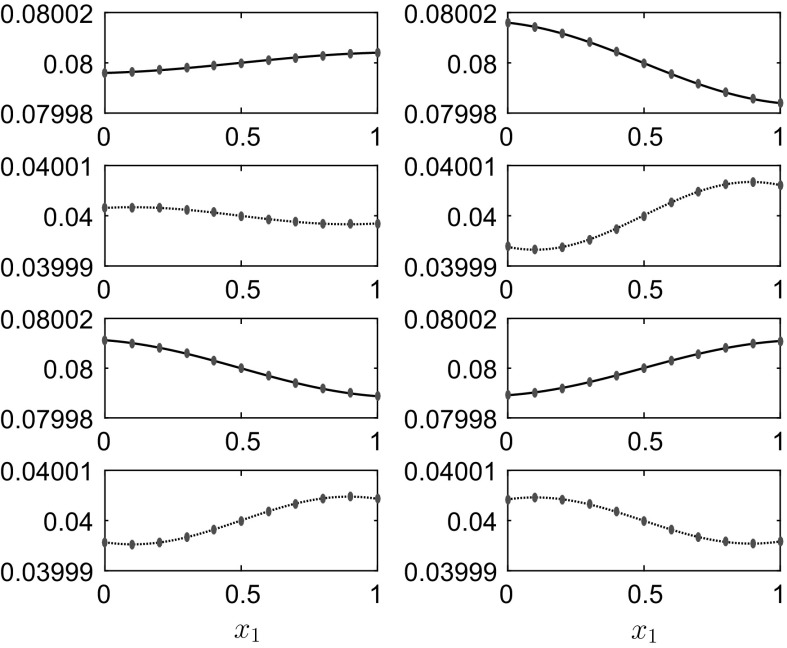
The interface positions $h_1(x_1,t)$ (*solid line* upper panels) and $h_1(x_1,t)+h_2(x_1,t)$ (*dotted line* lower panels) for the results in Fig. 4 at, *top* 2 *rows* $t=10$ and $t=20$, *bottom* 2 *rows* $t=30$ and $t=40$. The *dots* represent the exact linear solution (8.26) for the lowest-frequency mode. The rigid lid is at $h_1^0+h_2^0+h_3^0=0.12\,$m

**Fig. 15:**
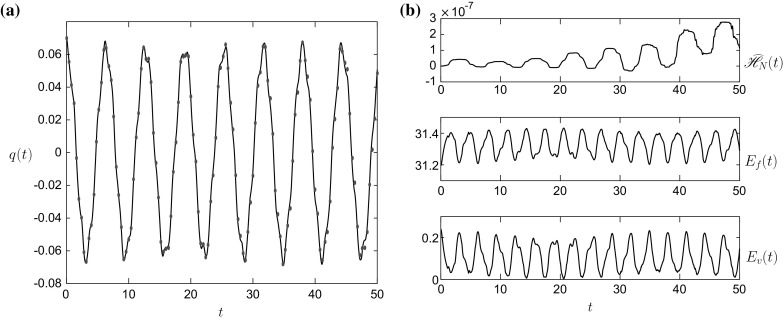
**a** The vessel displacement *q*(*t*) and **b** $E_f(t)$, $E_v(t)$ and $\widehat{\mathscr {H}}(t)$ for the nonlinear initial condition $Q_1=0$ and the initial parameter values given in column 2 of Table 2. The *dots* in represent the two-layer result given by Fig. 8

**Fig. 16:**
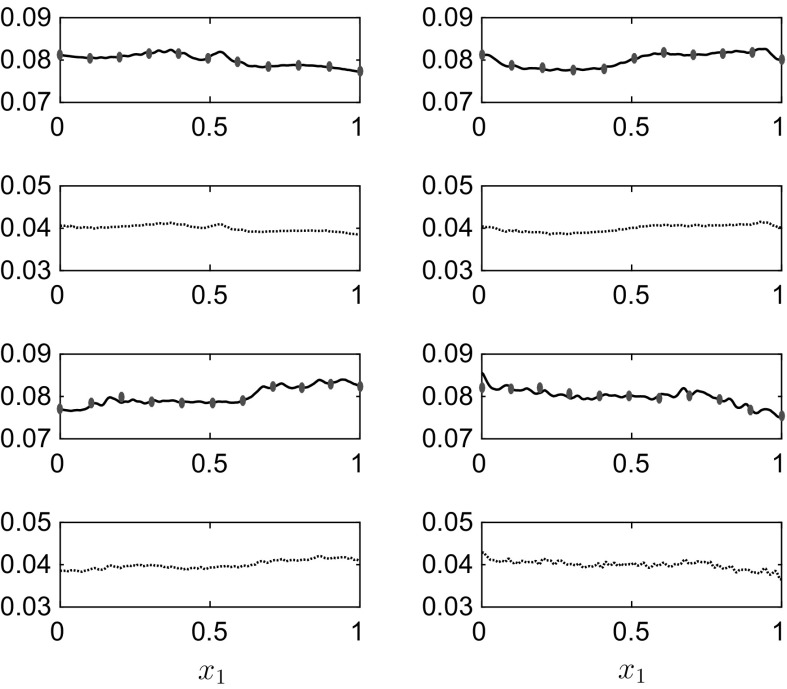
The interface positions $h_1(x_1,t)$ (*solid line* upper panels) and $h_1(x_1,t)+h_2(x_1,t)$ (*dotted line* lower panels) for the results in Fig. 8 at, *top* 2 *rows* $t=4$ and $t=8$, *bottom* 2 *rows* $t=18$ and $t=29$. The *dots* in the *upper panels* represent the two-layer result given by Fig. 9. The rigid lid is at $h_1^0+h_2^0+h_3^0=0.12\,$m

## Appendix 1: Equivalence of (2.17) and (2.23)

By using the definition of $w(a,\tau )$ in (2.20) one can show that (2.17) can be written as

6.1 $$\begin{aligned} -w_\tau =g(\rho _1-\rho _2)\frac{1}{x_{1a}}\left( \frac{\chi }{x_{1a}}\right) _a-\frac{\rho _2}{\chi }\left( \frac{d\chi x_{1\tau }x_{1a\tau }}{(dx_{1a}-\chi )^2}+\frac{d^2x_{1\tau }^2x_{1a}^2}{(dx_{1a}-\chi )^3}\left( \frac{\chi }{x_{1a}}\right) _a\right) . \end{aligned}$$

To show this is equivalent to (2.23), first note that

$$\begin{aligned} x_{1\tau }=\frac{Bw}{\chi (\rho _1-\rho _2)}-\frac{B}{A\chi }\left( p-I\right) , \end{aligned}$$

where $I=\int _0^LBw\,\mathrm{d}a$, and hence

$$\begin{aligned} x_{1a\tau }=\left( \frac{Bw}{\chi }\right) _a\frac{1}{(\rho _1-\rho _2)}-\left( \frac{B}{\chi }\right) _a\frac{\left( p-I\right) }{A}. \end{aligned}$$

Making these substitutions into (6.1) leads to

6.2 $$\begin{aligned} -w_{\tau }= & {} g(\rho _1-\rho _2)\frac{1}{x_{1a}}\left( \frac{\chi }{x_{1a}}\right) _a-d\rho _2\left[ \frac{1}{2(dx_{1a}-\chi )^2}\left[ \left( \frac{B^2w^2}{\chi ^2}\right) _a\frac{\chi }{(\rho _1-\rho _2)^2}\right. \right. \nonumber \\&\left. +\,\frac{2dx_{1a}^2}{dx_{1a}-\chi }\left( \frac{\chi }{x_{1a}}\right) _a\frac{B^2w^2}{\chi ^2(\rho _1-\rho _2)^2}\right] \nonumber \\&-\,\frac{(p-I)}{A}\left[ \frac{B}{(dx_{1a}-\chi )^2}\left( \frac{Bw}{\chi }\right) _a\frac{1}{\rho _1-\rho _2}+\frac{Bw}{\chi (\rho _1-\rho _2)}\left( \frac{B}{\chi }\right) _a\frac{1}{(dx_{1a}-\chi )^2}\right. \nonumber \\&\left. +\,\frac{2B^2w}{\chi ^2(\rho _1-\rho _2)}\frac{dx_{1a}^2}{(dx_{1a}-\chi )^3}\left( \frac{\chi }{x_{1a}}\right) _a\right] \nonumber \\&\left. +\,\frac{(p-I)^2}{A^2}\left[ \frac{B}{(dx_{1a}-\chi )^2}\left( \frac{B}{\chi }\right) _a+\frac{B^2}{\chi ^2}\frac{dx_{1a}^2}{(dx_{1a}-\chi )^3}\left( \frac{\chi }{x_{1a}}\right) _a\right] \right] . \end{aligned}$$

Now note that $\frac{\chi ^2}{\Gamma ^2}=\frac{B^2}{(dx_{1a}-\chi )^2(\rho _1-\rho _2)^2}$, hence

6.3 $$\begin{aligned} \left( \frac{\chi ^2\lambda }{\Gamma ^2}\right) _a =\frac{\chi }{(\rho _1-\rho _2)^2}\left[ \left( \frac{B^2\lambda }{\chi ^2}\right) _a\frac{\chi }{(dx_{1a}-\chi )^2}+2\left( \frac{B^2\lambda }{\chi ^2}\right) \frac{dx_{1a}^2}{(dx_{1a}-\chi )^3}\left( \frac{\chi }{x_{1a}}\right) _a\right] , \end{aligned}$$

where $\lambda$ represents *w* or $w^2$. Also,

$$\begin{aligned} \left( \frac{\chi ^2}{\Gamma ^2}\right) _a=\frac{2\chi }{(\rho _1-\rho _2)^2}\left[ \left( \frac{B}{\chi }\right) _a\frac{B}{(dx_{1a}-\chi )^2}+\frac{B^2}{\chi ^2}\frac{dx_{1a}^2}{(dx_{1a}-\chi )^3}\left( \frac{\chi }{x_{1a}}\right) _a\right] . \end{aligned}$$

Making these substitutions into (6.2) above, along with noting that

$$\begin{aligned} \frac{1}{x_{1a}}\left( \frac{\chi }{x_{1a}}\right) _a=\frac{1}{2\chi }\left( \frac{\chi ^2}{x_{1a}^2}\right) _a, \end{aligned}$$

gives the required form of (2.23).

## Appendix 2: Derivation of multilayer shallow-water equations with a rigid lid

In this section, we summarise the derivation of the multilayer shallow-water equations given in (4.1) and (4.2).

Consider the same *M*-layer system as considered in Sect. 4, so in the $i\mathrm{th}$ layer, the two-dimensional Euler equations are

$$\begin{aligned}&u_{it}+u_iu_{ix}+v_iu_{iy}+\rho _i^{-1}p_{ix}=-\ddot{q},\\&v_{it}+u_iv_{ix}+v_iv_{iy}+\rho _i^{-1}p_{iy}=-g,\\&u_{ix}+v_{iy}=0, \end{aligned}$$

where $i=1,...,M$, *x*,  *y*,  *t* subscripts denote partial derivatives and the over dots represent ordinary derivatives with respect to time. For simplicity, we drop the *i* subscripts from the layer coordinates $(x_i,y_i,t)$. In the shallow-water regime, the common assumptions on the flow field are that ${Dv_i}/{Dt}\approx 0$ and $u_{iy}=0$ [23]. Under these assumptions, the vertical momentum equation reduces to $p_{iy}=-g\rho _i$, which can be integrated from a general *y*-value in the layer to the upper-surface, denoted by $H_i=\sum _{j=1}^ih_j$, to give the pressure in each layer as

$$\begin{aligned} p_i(x,y,t)=P_i(x,t)+g\rho _i(H_i-y),~~~\hbox {for}~~~H_{i-1}\le y\le H_i. \end{aligned}$$

Here $P_i=p_i(x,H_i,t)$, $P_N=p_s(x,t)$ and $H_0=0$. Back substitution from the rigid lid to eliminate the $P_i$ expressions gives the $i^\mathrm{th}$ layer pressure as

$$\begin{aligned} p_i(x,y,t)=p_s+g\sum _{j=i+1}^M\rho _jh_j+g\rho _i\left( \sum _{j=1}^ih_j-y\right) ,~~~\hbox {for}~~~H_{i-1}\le y\le H_i. \end{aligned}$$

Substituting this into the horizontal momentum equation with $u_{iy}=0$ gives (4.2).

The conservation of mass equation is derived in the usual way by integrating the continuity equation across the fluid layer, noting that $u_{iy}=0$, so

$$\begin{aligned} 0=\int _{H_{i-1}}^{H_i}\left( u_{ix}+v_{iy}\right) \,\mathrm{d}y=\left. v_i\right| _{y=H_i}-\left. v_i\right| _{y=H_{i-1}}+(H_i-H_{i-1})u_{ix}, \end{aligned}$$

for $i=1,\cdots ,M$. Finally using the kinematic boundary condition on each interface and noting that $H_i-H_{i-1}=h_i$ leads to (4.1) for each layer *i*.

## Appendix 3: LPP Formulation for three layers

### Lagrangian formulation

From (4.1) and (4.2), the three-layer shallow-water equations are

8.1 $$\begin{aligned}&(\rho _1u_1)_t+\left( \frac{1}{2}\rho _1u_1^2+p_s+g\left[ \rho _1h_1+\rho _2h_2+\rho _3h_3\right] \right) _{x_1}=-\rho _1\ddot{q}, \end{aligned}$$

8.2 $$\begin{aligned}&(\rho _2u_2)_t+\left( \frac{1}{2}\rho _2u_2^2+p_s+g\left[ \rho _2h_1+\rho _2h_2+\rho _3h_3\right] \right) _{x_1}=-\rho _2\ddot{q}, \end{aligned}$$

8.3 $$\begin{aligned}&(\rho _3u_3)_t+\left( \frac{1}{2}\rho _3u_3^2+p_s+g\left[ \rho _3h_1+\rho _3h_2+\rho _3h_3\right] \right) _{x_1}=-\rho _3\ddot{q}, \end{aligned}$$

8.4 $$\begin{aligned}&h_{it}+(u_ih_i)_{x_1}=0,~~~\hbox {for}~~~i=1,2,3, \end{aligned}$$

where we have assumed the constraint that $x_1=x_2=x_3$. The interface thicknesses, $h_i(x_1,t)$, are constrained by the rigid lid constraint

8.5 $$\begin{aligned} h_1(x_1,t)+h_2(x_1,t)+h_3(x_1,t)=d. \end{aligned}$$

The motion of the vessel is governed by

8.6 $$\begin{aligned} \frac{\mathrm{d}}{\mathrm{d}t}\left[ \int _0^L\left( \rho _1h_1u_1+\rho _2h_2u_2+\rho _3h_3u_3\right) \,\mathrm{d}x_1+\widehat{M}\dot{q}\right] +\nu _1q-\nu _2q^3=0, \end{aligned}$$

where $\widehat{M}=m_v+m_f^{(1)}+m_f^{(2)}+m_f^{(3)}$  and $m_f^{(i)}=\int _0^L\rho _ih_i\,\mathrm{d}x_1$ is the fluid mass in the $i^\mathrm{th}$ layer.

Equation (4.5) gives the form for the *M*-layer shallow-water Lagrangian in the Eulerian framework. Thus, for the three-layer system this Lagrangian is

8.7 $$\begin{aligned} \mathscr {L}(h_1,h_2,h_3,u_1,u_2,u_3,q,\dot{q})=\int _{t_1}^{t_2}\int _0^L \widehat{L}\,\mathrm{d}x_1\mathrm{d}t+\int _{t_1}^{t_2}\left( \frac{1}{2}m_v\dot{q}^2-\frac{1}{2}\nu _1q^2+\frac{1}{4}\nu _2q^4\right) \,\mathrm{d}t, \end{aligned}$$

where

$$\begin{aligned} \widehat{L}= & {} \sum _{j=1}^3 L_j - g\left( \rho _2h_1h_2+\rho _3h_1h_3+\rho _3h_2h_3\right) -p_s\left( h_1+h_2+h_3-d\right) ,\\ L_j= & {} \frac{1}{2}\rho _jh_j\left( u_j+\dot{q}\right) ^2-\frac{1}{2}\rho _jgh_j^2+\rho _jf_j\left( h_{jt}+(h_ju_j)_{x_1}\right) . \end{aligned}$$

Note that $f_j$, $j=1,2,3$ and $p_s$ act as Lagrange multipliers. We can eliminate the top-layer variables $h_3$ and $u_3$ by using the constraint on the layer thicknesses (8.5) and the conservation of mass flux (4.6) which give

8.8 $$\begin{aligned} h_3=d-h_1-h_2~~~\hbox {and}~~~u_3=-\frac{u_1h_1+u_2h_2}{d-h_1-h_2}. \end{aligned}$$

To write the Lagrangian (8.7) in terms of the LPP formulation, we again consider the mapping (2.8) and we drop the subscript 1 on *a*, for simplicity. The constraint in the middle and upper layers become $x_1(a,\tau )=x_2(\phi _2(a,\tau ),\tau )$, and $x_1(a,\tau )=x_3(\phi _3(a,\tau ),\tau )$, where $\phi _2(a,\tau )$ and $\phi _3(a,\tau )$ are mappings. As for the two-layer case we do not have to consider the mapping function $\phi _3(a,\tau )$ because $h_3$ and $u_3$ are eliminated using (8.8). However, this time we do need to determine the mapping $\phi _2(a,\tau )$ as part of the solution procedure.

As was stated in Sect. 4, in the LPP formulation, we have

8.9 $$\begin{aligned} \begin{array}{cccccc} \widehat{h}_1(a,\tau )&{}=&{}\dfrac{\chi _1(a)}{x_{1a}},&{}~~~~\widehat{h}_2(a,\tau )&{}=&{}\dfrac{\chi _2(\phi _2)\phi _{2a}}{x_{1a}},\\ u_1(a,\tau )&{}=&{}x_{1\tau },&{}~~~~u_2(a,\tau )&{}=&{}x_{1\tau }-\dfrac{x_{1a}\phi _{2\tau }}{\phi _{2a}}. \end{array} \end{aligned}$$

Substituting these into the Lagrangian (8.7) and manipulating the terms leads to

8.10 $$\begin{aligned} \mathscr {L}=\int _{t_1}^{t_2}\int _0^L \widetilde{L}\,\mathrm{d}a\mathrm{d}t+\int _{t_1}^{t_2}\left( \frac{1}{2}m_v\dot{q}^2-\frac{1}{2}\nu _1q^2+\frac{1}{4}\nu _2q^4\right) \,\mathrm{d}t, \end{aligned}$$

where

$$\begin{aligned} \widetilde{L}= & {} \frac{1}{2}\widehat{\alpha }x_{1\tau }^2+\frac{1}{2}\widehat{\gamma }\phi _{2\tau }^2-\widehat{\beta }x_{1\tau }\phi _{2\tau }+\widehat{\sigma }_1x_{1\tau }q_\tau +\widehat{\sigma }_2\phi _{2\tau }q_\tau \\&+\,\frac{1}{2}\left( \rho _1\chi _1+\rho _2\chi _2\phi _{2a}+\rho _3(dx_{1a}-\chi _1-\chi _2\phi _{2a})\right) q_\tau ^2\\&-\,\frac{g}{2x_{1a}}\left[ \widetilde{\rho }_{[1,3]}\chi _1^2+\widetilde{\rho }_{[2,3]}\chi _2^2\phi _{2a}^2+2\widetilde{\rho }_{[2,3]}\chi _1\chi _2\phi _{2a}\right] -\frac{1}{2}g\rho _3d^2x_{1a}. \end{aligned}$$

and

$$\begin{aligned} \widehat{\alpha }= & {} \rho _1\chi _1+\rho _2\chi _2\phi _{2a}+\frac{\rho _3(\chi _1+\chi _2\phi _{2a})^2}{dx_{1a}-\chi _1-\chi _2\phi _{2a}},\\ \widehat{\beta }= & {} \chi _2x_{1a}\left[ \rho _2+\frac{\rho _3(\chi _1+\chi _2\phi _{2a})}{dx_{1a}-\chi _1-\chi _2\phi _{2a}}\right] ,\\ \widehat{\gamma }= & {} \frac{\chi _2x_{1a}^2}{\phi _{2a}}\left[ \rho _2+\frac{\rho _3\chi _2\phi _{2a}}{dx_{1a}-\chi _1-\chi _2\phi _{2a}}\right] ,\\ \widehat{\sigma }_1= & {} \widetilde{\rho }_{[1,3]}\chi _1+\widetilde{\rho }_{[2,3]}\chi _2\phi _{2a},\\ \widehat{\sigma }_2= & {} -\widetilde{\rho }_{[2,3]}\chi _2x_{1a},\\ \widetilde{\rho }_{[1,2]}= & {} \rho _1-\rho _2,~~\widetilde{\rho }_{[2,3]}=\rho _2-\rho _3,~~\widetilde{\rho }_{[1,3]}=\rho _1-\rho _3. \end{aligned}$$

At this stage it is also worth noting that

$$\begin{aligned} \int _0^L\rho _1\chi _1\,\mathrm{d}a=m_f^{(1)},~~~\int _0^L\rho _2\chi _2\phi _{2a}\,\mathrm{d}a=m_f^{(2)},~~~\int _0^L\rho _3(dx_{1a}-\chi _1-\chi _2\phi _{2a})\,\mathrm{d}a=m_f^{(3)}. \end{aligned}$$

### Hamiltonian formulation

The coupled system Lagrangian (8.10) can be written in terms of a Hamiltonian functional with canonical variables $(x_1,\phi _2,q,w_1,w_2,p)$, where

8.11 $$\begin{aligned}&w_1(a,\tau )=\frac{\delta \mathscr {L}}{\delta x_{1\tau }}=\widehat{\alpha }x_{1\tau }-\widehat{\beta }\phi _{2\tau }+\widehat{\sigma }_1q_\tau , \end{aligned}$$

8.12 $$\begin{aligned}&w_2(a,\tau )=\frac{\delta \mathscr {L}}{\delta \phi _{2\tau }}=-\widehat{\beta }x_{1\tau }+\widehat{\gamma }\phi _{2\tau }+\widehat{\sigma }_2q_\tau , \end{aligned}$$

8.13 $$\begin{aligned}&p(\tau )=\frac{\delta \mathscr {L}}{\delta q_\tau }=\int _0^L\left[ \widehat{\sigma }_1x_{1\tau }+\widehat{\sigma }_2\phi _{2\tau }\right] \,\mathrm{d}a+\widehat{M}q_\tau . \end{aligned}$$

To write the Hamiltonian in terms of the canonical variables, we need to eliminate $x_{1\tau }$, $\phi _{2\tau }$ and $q_\tau$ from the Lagrangian and the resulting Hamiltonian. Inverting (8.11)–(8.13) gives

$$\begin{aligned} x_{1\tau }=C_1w_1+C_3w_2-B_1q_\tau ,~~\phi _{2\tau }=C_3w_1+C_2w_2-B_2q_\tau ,~~q_\tau =\frac{(p-I)}{A}, \end{aligned}$$

where

$$\begin{aligned} C_1= & {} \frac{\widehat{\gamma }}{\widehat{\alpha }\widehat{\gamma }-\widehat{\beta }^2},~~C_2=\frac{\widehat{\alpha }}{\widehat{\alpha }\widehat{\gamma }-\widehat{\beta }^2},~~C_3=\frac{\widehat{\beta }}{\widehat{\alpha }\widehat{\gamma }-\widehat{\beta }^2},\\ B_1= & {} C_1\widehat{\sigma }_1+C_3\widehat{\sigma }_2,~~B_2=C_3\widehat{\sigma }_1+C_2\widehat{\sigma }_2,\\ I= & {} \int _0^L\left( B_1w_1+B_2w_2\right) \,\mathrm{d}a,\\ A= & {} M-\int _0^LA_1\,\mathrm{d}a,~~~\hbox {with}~~~A_1=C_1\widehat{\sigma }_1^2+2C_3\widehat{\sigma }_1\widehat{\sigma }_2+C_2\widehat{\sigma }_2^2. \end{aligned}$$

Therefore using the above definitions and taking the Legendre transformation of (8.10) leads to

8.14 $$\begin{aligned} \mathscr {H}(x_1,\phi _2,q,w_1,w_2,p)= & {} \frac{1}{2}\int _0^L\left[ C_1w_1^2+C_2w_2^2+2C_3w_1w_2\right] \,\mathrm{d}a+\frac{1}{2A}\left( p-I\right) ^2,\nonumber \\&+\int _0^L\frac{g}{2x_{1a}}\left[ \widetilde{\rho }_{[1,3]}\chi _1^2+\widetilde{\rho }_{[2,3]}\chi _2^2\phi _{2a}^2+2\widetilde{\rho }_{[2,3]}\chi _1\chi _2\phi _{2a}\right] \,\mathrm{d}a\nonumber \\&+\frac{1}{2}\rho _3gd^2L+\frac{1}{2}\nu _1q^2-\frac{1}{4}\nu _2q^4, \end{aligned}$$

where the functions $C_1,~C_2,~C_3$, $B_1$ and $B_2$ simplify to

$$\begin{aligned} C_1= & {} \frac{1}{\chi _1\rho _1}\left( 1-\frac{\rho _2\rho _3\chi _1}{\Gamma }\right) ,\\ C_2= & {} \frac{\phi _{2a}}{\chi _1\chi _2x_{1a}^2}\left( \frac{\chi _1}{\rho _2}+\frac{\chi _2\phi _{2a}}{\rho _1}-\frac{\rho _3\widetilde{\rho }_{[1,2]}^2\chi _1\chi _2\phi _{2a}}{\rho _1\rho _2\Gamma }\right) ,\\ C_3= & {} \frac{\phi _{2a}}{\chi _1x_{1a}\rho _1}\left( 1+\frac{\rho _3\widetilde{\rho }_{[1,2]}\chi _1}{\Gamma }\right) ,\\ B_1= & {} 1-\frac{\rho _2\rho _3dx_{1a}}{\Gamma },\\ B_2= & {} \frac{\rho _3\widetilde{\rho }_{[1,2]}d\phi _{2a}}{\Gamma },\\ \Gamma= & {} \rho _1\rho _2(dx_{1a}-\chi _1-\chi _2\phi _{2a})+\rho _1\rho _3\chi _2\phi _{2a}+\rho _2\rho _3\chi _1. \end{aligned}$$

Taking variations of the Hamiltonian with respect to the six conical variables gives Hamilton’s equations

8.15 $$\begin{aligned} -w_{1\tau }=\frac{\delta \mathscr {H}}{\delta x_1}= & {} -\frac{1}{2}d\rho _2^2\rho _3\left( \frac{w_1^2}{\Gamma ^2}\right) _a\nonumber \\&+\,\frac{1}{2}\left[ \frac{2}{\rho _2}\left( \frac{\phi _{2a}w_2^2}{\chi _2x_{1a}^3}\right) _a+\frac{2}{\rho _1}\left( \frac{\phi _{2a}^2w_2^2}{\chi _1x_{1a}^3}\right) _a-\frac{\rho _3\widetilde{\rho }_{[1,2]}^2}{\rho _1\rho _2}\left( \frac{2\phi _{2a}^2w_2^2}{\Gamma x_{1a}^3}+\frac{d\rho _1\rho _2\phi _{2a}^2w_2^2}{x_{1a}^2\Gamma ^2}\right) _a\right] \nonumber \\&+\,\frac{1}{\rho _1}\left( \frac{\phi _{2a}w_1w_2}{\chi _1x_{1a}^2}\right) _a+\frac{\rho _3\widetilde{\rho }_{[1,2]}}{\rho _1}\left( \frac{\phi _{2a}w_1w_2}{x_a^2\Gamma }+\frac{d\rho _1\rho _2\phi _{2a}w_1w_2}{x_{1a}\Gamma ^2}\right) _a\nonumber \\&-\,\frac{(p-I)}{A}\left[ d\rho _2\rho _3\left[ \left( \frac{w_1}{\Gamma }\right) _a-d\rho _1\rho _2\left( \frac{x_{1a}w_1}{\Gamma ^2}\right) _a\right] +d^2\rho _1\rho _2\rho _3\widetilde{\rho }_{[1,2]}\left( \frac{\phi _{2a}w_2}{\Gamma ^2}\right) _a\right] \nonumber \\&-\,\frac{(p-I)^2}{2A^2}d\rho _3\left( \frac{[\rho _2\widetilde{\rho }_{[1,3]}\chi _1+\rho _1\widetilde{\rho }_{[2,3]}\chi _2\phi _{2a}]^2}{\Gamma ^2}\right) _a\nonumber \\&+\,\frac{g}{2}\left( \frac{\widetilde{\rho }_{[1,3]}\chi _1^2+\widetilde{\rho }_{[2,3]}\chi _2^2\phi _{2a}^2+2\widetilde{\rho }_{[2,3]}\chi _1\chi _2\phi _{2a}}{x_{1a}^2}\right) _a, \end{aligned}$$

8.16 $$\begin{aligned} -w_{2\tau }=\frac{\delta \mathscr {H}}{\delta \phi _2}= & {} \frac{1}{2}\rho _2\rho _3\widetilde{\rho }_{[2,3]}\left( \frac{\chi _2w_1^2}{\Gamma ^2}\right) _a-\frac{1}{2}\left[ \frac{1}{\rho _2}\left( \frac{w_2^2}{\chi _2x_{1a}^2}\right) _a+\frac{2}{\rho _1}\left( \frac{\phi _{2a}w_2^2}{\chi _1x_{1a}^2}\right) _a\right. \nonumber \\&\left. -\,\frac{\rho _3\widetilde{\rho }_{[1,2]}^2}{\rho _1\rho _2}\left[ 2\left( \frac{\phi _{2a}w_2^2}{x_{1a}^2\Gamma }\right) _a+\rho _1\widetilde{\rho }_{[2,3]}\left( \frac{\chi _2\phi _{2a}^2w_2^2}{x_{1a}^2\Gamma ^2}\right) _a\right] \right] \nonumber \\&-\frac{1}{\rho _1}\left( \frac{w_1w_2}{\chi _1x_{1a}}\right) _a-\frac{\rho _3\widetilde{\rho }_{[1,2]}}{\rho _1}\left[ \left( \frac{w_1w_2}{x_{1a}\Gamma }\right) _a+\rho _1\widetilde{\rho }_{[2,3]}\left( \frac{\chi _2\phi _{2a}w_1w_2}{x_{1a}\Gamma ^2}\right) _a\right] \nonumber \\&-\,\frac{(p-I)}{A}\left[ d\rho _1\rho _2\rho _3\widetilde{\rho }_{[2,3]}\left( \frac{\chi _2x_{1a}w_1}{\Gamma ^2}\right) _a\right. \nonumber \\&\left. -\,d\rho _3\widetilde{\rho }_{[1,2]}\left( \frac{w_2}{\Gamma }+\rho _1\widetilde{\rho }_{[2,3]}\frac{\chi _2\phi _{2a}w_2}{\Gamma ^2}\right) _a\right] -\frac{(p-I)^2}{2A^2}\left[ \frac{\widetilde{\rho }_{[2,3]}^2}{\rho _2}\left( \chi _2\right) _a\right. \nonumber \\&-\,\frac{\rho _3}{\rho _1\rho _2}\left( 2\rho _1\widetilde{\rho }_{[2,3]}\chi _2\frac{[\rho _2\widetilde{\rho }_{[1,3]}\chi _1+\rho _1\widetilde{\rho }_{[2,3]}\chi _2\phi _{2a}]}{\Gamma }\right. \nonumber \\&\left. \left. +\rho _1\widetilde{\rho }_{[2,3]}\chi _2\frac{[\rho _2\widetilde{\rho }_{[1,3]}\chi _1+\rho _1\widetilde{\rho }_{[2,3]}\chi _2\phi _{2a}]^2}{\Gamma ^2}\right) _a\right] \nonumber \\&-\,g\widetilde{\rho }_{[2,3]}\left[ \left( \frac{\chi _1\chi _2}{x_{1a}}\right) _a+\left( \frac{\chi _2^2\phi _{2a}}{x_{1a}}\right) _a\right] , \end{aligned}$$

8.17 $$\begin{aligned} -p_\tau =\frac{\delta \mathscr {H}}{\delta q}= & {} \nu _1q-\nu _2q^3, \end{aligned}$$

8.18 $$\begin{aligned} x_{1\tau }=\frac{\delta \mathscr {H}}{\delta w_1}= & {} C_1w_1+C_3w_2-\frac{(p-I)}{A}B_1, \end{aligned}$$

8.19 $$\begin{aligned} \phi _{2\tau }=\frac{\delta \mathscr {H}}{\delta w_2}= & {} C_2w_2+C_3w_1-\frac{(p-I)}{A}B_2, \end{aligned}$$

8.20 $$\begin{aligned} q_\tau =\frac{\delta \mathscr {H}}{\delta p}= & {} \frac{(p-I)}{A}. \end{aligned}$$

### Linear solution

The linear solution of the three-layer shallow-water scheme will be used to validate the numerical method. The linear solution here can be derived in one of two ways. One could solve the linearised form of Hamilton’s equations (8.15)–(8.20) or one could solve the linearised the Eulerian shallow-water equations (8.1)–(8.4). Here we choose the latter and then convert the solutions into the LPP framework at the end.

We seek a linear solution to (8.1)–(8.4) about a quiescent fluid with lower, middle and upper mean layer thicknesses $h_1^0$, $h_2^0$ and $h_3^0$, respectively, of the form

$$\begin{aligned} u_i(x,t)=\widehat{U}_i(x,t),\quad \hbox {and}\quad h_i(x,t)=h_i^0+\widehat{H}_i(x,t)\quad \hbox {for}~~i=1,2,3, \end{aligned}$$

where $|\widehat{U}_i|\ll 1, |\widehat{H}_i|\ll 1$ and $|q|\ll 1$ and $p_s=p_s^{0}+\widehat{P}(x,t)$ (Note that we have dropped the suffix 1 from the *x* for simplicity). Substituting these expressions into the shallow-water equations (8.1)–(8.4) leads to

8.21 $$\begin{aligned}&\widehat{U}_{1t}+g\left( \widehat{H}_{1x}+\frac{\rho _2}{\rho _1}\widehat{H}_{2x}+\frac{\rho _3}{\rho _1}\widehat{H}_{3x}\right) +\frac{1}{\rho _1}\widehat{P}_x=-\ddot{q}, \end{aligned}$$

8.22 $$\begin{aligned}&\widehat{U}_{2t}+g\left( \widehat{H}_{1x}+\widehat{H}_{2x}+\frac{\rho _3}{\rho _2}\widehat{H}_{3x}\right) +\frac{1}{\rho _2}\widehat{P}_x=-\ddot{q}, \end{aligned}$$

8.23 $$\begin{aligned}&\widehat{U}_{3t}+g\left( \widehat{H}_{1x}+\widehat{H}_{2x}+\widehat{H}_{3x}\right) +\frac{1}{\rho _3}\widehat{P}_x=-\ddot{q}, \end{aligned}$$

8.24 $$\begin{aligned}&\widehat{H}_{it}+h_i^0\widehat{U}_{ix}=0,~~~i=1,2,3, \end{aligned}$$

and the constraint (8.8) gives

8.25 $$\begin{aligned} h_1^0+h_2^0+h_3^0=d,\quad \hbox {and}\quad \widehat{H}_1+\widehat{H}_2+\widehat{H}_3=0. \end{aligned}$$

Seeking a harmonic solutions to (8.21)–(8.25) with frequency $\omega$, such that

8.26 $$\begin{aligned} \widehat{U}_i(x,t)=U(x)\sin (\omega t),~~~q(t)=Q\cos (\omega t),~~~H_i(x,t)=H(x)\cos (\omega t), \end{aligned}$$

for $i=1,2,3$, leads to

8.27 $$\begin{aligned}&\omega U_1+g\left( H_1'+\frac{\rho _2}{\rho _1}H_2'+\frac{\rho _3}{\rho _1}H_3'\right) +\frac{P'}{\rho _1}=\omega ^2Q, \end{aligned}$$

8.28 $$\begin{aligned}&\omega U_2+g\left( H_1'+H_2'+\frac{\rho _3}{\rho _2}H_3'\right) +\frac{P'}{\rho _2}=\omega ^2Q, \end{aligned}$$

8.29 $$\begin{aligned}&\omega U_3+\frac{P'}{\rho _3}=\omega ^2Q, \end{aligned}$$

8.30 $$\begin{aligned}&H_i=\frac{h_i^0}{\omega }U_i',~~\hbox {for}~~i=1,2,3. \end{aligned}$$

Note from the conservation of flux equation (8.8) that $U_3=-\frac{1}{h_3^0}\left( h_1^0U_1+h_2^0U_2\right)$. Eliminating $H_i$ for $i=1,2,3$, $U_3$ and *P* from (8.27)–(8.30) leads to the first two momentum equations being written as

8.31 $$\begin{aligned} \omega \left[ \left( \rho _1+\frac{\rho _3h_1^0}{h_3^0}\right) U_1+\frac{\rho _3h_2^0}{h_3^0}U_2\right] +\frac{g}{\omega }\left[ h_1^0\widetilde{\rho }_{[1,3]}U_1''+h_2^0\widetilde{\rho }_{[2,3]}U_2''\right]= & {} \omega ^2\widetilde{\rho }_{[1,3]}Q, \end{aligned}$$

8.32 $$\begin{aligned} \omega \left[ \frac{\rho _3h_1^0}{h_3^0}U_1+\left( \rho _2+\frac{\rho _3h_2^0}{h_3^0}\right) U_2\right] +\frac{g}{\omega }\left[ h_1^0\widetilde{\rho }_{[2,3]}U_1''+h_2^0\widetilde{\rho }_{[2,3]}U_2''\right]= & {} \omega ^2\widetilde{\rho }_{[2,3]}Q. \end{aligned}$$

Eliminating $U_2''$ from these two equations leads to an expression for $U_2$

8.33 $$\begin{aligned} U_2=\frac{\rho _1}{\rho _2}U_1+\frac{gh_1^0}{\rho _2\omega ^2}\widetilde{\rho }_{[1,2]}U_1''-\frac{\omega }{\rho _2}\widetilde{\rho }_{[1,2]}Q, \end{aligned}$$

which can be substituted into (8.31) giving the $4\mathrm{th}$ order ODE for $U_1$

8.34 $$\begin{aligned} g^2\xi _1U_1''''+\omega ^2g\xi _2U_1''+\omega ^4\xi _3U_1=\omega ^5\xi _4Q, \end{aligned}$$

where

$$\begin{aligned}&\xi _1=h_1^0h_2^0h_3^0\widetilde{\rho }_{[1,2]}\widetilde{\rho }_{[2,3]},\quad \xi _2=h_1^0h_2^0\rho _3\widetilde{\rho }_{[1,2]}+h_2^0h_3^0\rho _1\widetilde{\rho }_{[2,3]}+h_1^0h_3^0\rho _2\widetilde{\rho }_{[1,3]},\\&\xi _3=h_1^0\rho _2\rho _3+h_2^0\rho _1\rho _3+h_3^0\rho _1\rho _2,~ \xi _4=h_2^0\rho _3\widetilde{\rho }_{[1,2]}+h_3^0\rho _2\widetilde{\rho }_{[1,3]}. \end{aligned}$$

The general solution to this ODE is

8.35 $$\begin{aligned} U_1(x)= & {} \alpha _1\cos \left( \frac{\omega }{g^{1/2}}Z_1x\right) +\alpha _2\sin \left( \frac{\omega }{g^{1/2}}Z_1x\right) \nonumber \\&\quad +\,\alpha _3\cos \left( \frac{\omega }{g^{1/2}}Z_2x\right) +\alpha _4\sin \left( \frac{\omega }{g^{1/2}}Z_2x\right) +\frac{\xi _4}{\xi _3}\omega Q, \end{aligned}$$

where $\lambda \in \{\pm \mathrm{i}Z_1,\pm \mathrm{i}Z_2\}$ ($\mathrm{i}=\sqrt{-1}$) are roots of the quartic equation

$$\begin{aligned} \xi _1\lambda ^4+\xi _2\lambda ^2+\xi _3=0. \end{aligned}$$

The four unknown constants, $\alpha _i$, are found from the four boundary conditions $u_1=u_2=0$ at $x=0,L$. In terms of the function $U_1$, these conditions are

$$\begin{aligned} U_1(0)=U_1(L)=0~~~\hbox {and}~~~U_1''(0)=U_1''(L)=\frac{\omega ^3Q}{gh_1^0}, \end{aligned}$$

where (8.33) has been used in deriving the second set of boundary conditions. Inserting (8.35) into the above boundary conditions gives

$$\begin{aligned} \alpha _1= & {} \omega Q\frac{\frac{\xi _4}{\xi _3}Z_2^2-\frac{1}{h_1^0}}{Z_1^2-Z_2^2},~~~~~~\alpha _2=\omega Q\frac{\frac{\xi _4}{\xi _3}Z_2^2-\frac{1}{h_1^0}}{Z_1^2-Z_2^2}\tan \left( \frac{\omega }{2g^{1/2}}Z_1L\right) ,\\ \alpha _3= & {} -\omega Q\frac{\frac{\xi _4}{\xi _3}Z_1^2-\frac{1}{h_1^0}}{Z_1^2-Z_2^2},~~~~~\alpha _4=-\omega Q\frac{\frac{\xi _4}{\xi _3}Z_1^2-\frac{1}{h_1^0}}{Z_1^2-Z_2^2}\tan \left( \frac{\omega }{2g^{1/2}}Z_2L\right) . \end{aligned}$$

Note that in solving for $\alpha _1$ to $\alpha _4$ we have assumed that the solutions are away from any resonance, i.e. we have neglected solutions with $\sin (\frac{\omega }{2g^{1/2}}Z_iL)=0$ for $i=1$ or 2. Thus,

8.36 $$\begin{aligned} U_1(x)= & {} \widehat{\alpha }_1\widehat{f}_1(x)-\widehat{\alpha }_2\widehat{f}_2(x)+\frac{\xi _4}{\xi _3}\omega Q, \end{aligned}$$

8.37 $$\begin{aligned} U_2(x)= & {} -\widehat{\alpha }_1\widehat{f}_1(x)\left( \frac{h_1^0Z_1^2\widetilde{\rho }_{[1,2]}-\rho _1}{\rho _2}\right) +\widehat{\alpha }_2\widehat{f}_2(x)\left( \frac{h_1^0Z_2^2\widetilde{\rho }_{[1,2]}-\rho _1}{\rho _2}\right) +\frac{\rho _1\xi _4}{\rho _2\xi _3}\omega Q-\frac{\omega }{\rho _2}\widetilde{\rho }_{[1,2]}Q, \end{aligned}$$

8.38 $$\begin{aligned} U_3(x)= & {} -\frac{1}{h_3^0}\left[ \widehat{\alpha }_1\widehat{f}_1(x)\left( \frac{h_1^0\rho _2+h_2^0\rho _1-h_1^0h_2^0Z_1^2\widetilde{\rho }_{[1,2]}}{\rho _2}\right) -\widehat{\alpha }_2\widehat{f}_2(x)\left( \frac{h_1^0\rho _2+h_2^0\rho _1-h_1^0h_2^0Z_2^2\widetilde{\rho }_{[1,2]}}{\rho _2}\right) \right. \nonumber \\&\left. +\frac{\xi _4(h_1^0\rho _2+h_2^0\rho _1)}{\xi _3\rho _2}\omega Q-\frac{\omega h_2^0}{\rho _2}\widetilde{\rho }_{[1,2]}Q\right] , \end{aligned}$$

where $\widehat{f}_i=\cos (\frac{\omega }{g^{1/2}}Z_ix)+\tan (\frac{\omega }{2g^{1/2}}Z_iL)\sin (\frac{\omega }{g^{1/2}}Z_ix),~~\hbox {for}~~i=1,2$, and $\widehat{\alpha }_1=\frac{\alpha _1}{\omega Q}$ and $\widehat{\alpha }_2=\frac{\alpha _3}{\omega Q}$.

To find the characteristic frequencies of the system, we use the linearised version of the vessel equation (8.6)

8.39 $$\begin{aligned} \omega \left[ \int _0^L\rho _1h_1^0\widehat{u}_1+\rho _2h_2^0\widehat{u}_2+\rho _3h_3^0\widehat{u}_3\,\mathrm{d}x-\omega \widehat{M}Q\right] +\nu _1Q=0. \end{aligned}$$

Substituting in the harmonic forms (8.26) and noting that $\int _0^L \widehat{f}_i(x)\,\mathrm{d}x=\frac{2g^{1/2}}{\omega Z_i}\tan (\frac{\omega }{2g^{1/2}}Z_iL)$, leads to the characteristic equation

8.40 $$\begin{aligned}&\nu _1-\widehat{M}\omega ^2+\frac{L\omega ^2}{\rho _2}\left[ \left( h_1^0\rho _2\widetilde{\rho }_{[1,3]}+h_2^0\rho _1\widetilde{\rho }_{[2,3]}\right) \frac{\xi _4}{\xi _3}-h_2^0\widetilde{\rho }_{[1,2]}\widetilde{\rho }_{[2,3]}\right] \nonumber \\&\quad -\,\frac{2g^{1/2}\widehat{\alpha }_1\omega }{\rho _2Z_1}\left[ Z_1^2h_1^0h_2^0\widetilde{\rho }_{[1,2]}\widetilde{\rho }_{[2,3]}-h_1^0\rho _2\widetilde{\rho }_{[1,3]}-h_2^0\rho _1\widetilde{\rho }_{[2,3]}\right] \tan \left( \frac{\omega }{2g^{1/2}}Z_1L\right) \nonumber \\&\quad +\,\frac{2g^{1/2}\widehat{\alpha }_2\omega }{\rho _2Z_2}\left[ Z_2^2h_1^0h_2^0\widetilde{\rho }_{[1,2]}\widetilde{\rho }_{[2,3]}-h_1^0\rho _2\widetilde{\rho }_{[1,3]}-h_2^0\rho _1\widetilde{\rho }_{[2,3]}\right] \tan \left( \frac{\omega }{2g^{1/2}}Z_2L\right) =0, \end{aligned}$$

which is solved via Newton iterations for the roots $\omega$.

The Eulerian velocities are converted into Lagrangian velocities to give initial conditions for the LPP problem by using (8.9), and thus, in the linear regime

8.41 $$\begin{aligned} x_1(a,\tau )= & {} a-\frac{U_1(a)}{\omega }\cos (\omega \tau ), \end{aligned}$$

8.42 $$\begin{aligned} \phi _2(a,\tau )= & {} a+\frac{U_2(a)-U_1(a)}{\omega }\cos (\omega \tau ), \end{aligned}$$

8.43 $$\begin{aligned} \widehat{h}_i(a,\tau )= & {} h_i^0+\frac{h_i^0}{\omega }U_i'(a)\cos (\omega \tau ),~~\hbox {for}~~i=1,2. \end{aligned}$$

### Numerical algorithm

To discretize the equations (8.15)–(8.20), we use the same approach as for the two-layer problem. Thus, we discretize the Lagrangian state space into *N* parcels using (3.1) and let $x_i(t):=x_1(a_i,\tau )$, $\phi _i(t):=\phi _2(a_i,\tau )$, $w_{1i}(t):=w_1(a_i,\tau )$ and $w_{2i}(t):=w_2(a_i,\tau )$. We again use the semi-discretization method to derive the resulting discretized form of Hamilton’s equations and rather than write out the whole form of the discretized equations, we note that they can be derived from (8.15)–(8.20) by noting $(\mu )_a:=\frac{1}{\Delta a}(\mu _i-\mu _{i-1})$, in the semi-discretization. See (2.23) and (3.3).

The discretized system requires 8 boundary conditions. Four are

$$\begin{aligned} x_1=0,~~x_{N+1}=L,~~\phi _1=0,~~\phi _{N+1}=L, \end{aligned}$$

and as these lead to $(x_1)_\tau =(x_{N+1})_\tau =(\phi _1)_\tau =(\phi _{N+1})_\tau =0$, then from (8.18) and (8.19) the other four boundary conditions are

$$\begin{aligned} w_{1j}=\frac{(p-I)}{A(C_{1j}C_{2j}-C_{3j}^2)}\left( C_{2j}B_{1j}-C_{3j}B_{2j}\right) ,~~w_{2j}=\frac{(p-I)}{A(C_{1j}C_{2j}-C_{3j}^2)}\left( C_{1j}B_{2j}-C_{3j}B_{1j}\right) , \end{aligned}$$

for $$j=1$$ and $$N+1$$.

The discretized set of equations and their boundary conditions can be written in the form (3.7) where $\mathbf{p}=(p,w_{1},\ldots ,w_{1(N+1)},w_{21},\ldots ,w_{2(N+1)})$ and $\mathbf{q}=(q,x_1,\ldots ,x_{N+1},\phi _1,\ldots ,\phi _{N+1})$. These equations are time discretized using the *implicit-midpoint rule* and the resulting system of $4N+6$ nonlinear algebraic equations are solved via Newton iterations.

The initial conditions used to validate of the scheme are the linear forms from Sects. (8.41)–(8.43)

$$\begin{aligned}&q(0)=Q,~~q_\tau (0)=0,~~x_1(a,0)=a-\frac{U_1(a)}{\omega }Q_1,~~\phi _2(a,0)=a+\frac{U_2(a)-U_1(a)}{\omega }Q_1,\\&\widehat{h}_1(a,0)=h_{1}^{0}+\frac{h_1^0}{\omega }U_1'(a)Q_1,~~\widehat{h}_2(a,0)=h_{2}^{0}+\frac{h_2^0}{\omega }U_2'(a)Q_1,\\&w_1(a,0)=w_2(a,0)=p(0)=0, \end{aligned}$$

where $U_1(a)$ and $U_2(a)$ are given by (8.36) and (8.37) with *x* replaced by *a*, and $Q_1$ is an independent parameter. When $Q_1=1$, the initial condition is that given by the linear problem, while when $Q_1=0$ it is an initial condition achievable in an experiment, namely a horizontally displaced vessel released from rest with a quiescent fluid.

### Numerical scheme validation results

For the validation results presented here, we set $N=200$ and $\Delta \tau =10^{-3}$ for the linear simulation and $\Delta \tau =10^{-4}$ for the nonlinear simulation. The parameter values for the two simulations are given in Table 2.

**Table 2:** Simulation parameter values for Figs. 13, 14, 15 and 16

Parameter (units)	Figs. 13, 14	Figs. 15, 16
*L* (m)	1	1
*d* (m)	0.12	0.12
$h_1^0$ (m)	0.04	0.04
$h_2^0$ (m)	0.04	0.04
$h_3^0$ (m)	0.04	0.04
$\rho _1$ ($\mathrm{kg\,m}^{-3}$)	1000	1000
$\rho _2$ ($\mathrm{kg\,m}^{-3}$)	500	990
$\rho _3$ ($\mathrm{kg\,m}^{-3}$)	100	1
$m_f^{(1)}$ (kg)	40	40
$m_f^{(2)}$ (kg)	20	39.6
$m_f^{(3)}$ (kg)	4	0.04
$m_v$ (kg)	10	10
*Q* (m)	$10^{-4}$	0.07
$\nu _1$ ($\mathrm{kg\,s}^{-2}$)	100	100
$\nu _2$ ($\mathrm{kg\,m}^{-2}\mathrm{s}^{-2}$)	0	800
$\omega$ ($\mathrm{s}^{-1}$)	1.1224	–

Note that for the three-fluid system, the vessel energy is given by

$$\begin{aligned} E_v=\frac{m_v}{2A^2}\left( p-I\right) ^2+\frac{1}{2}\nu _1q^2-\frac{1}{4}\nu _2q^4, \end{aligned}$$

while the fluid energy is

$$\begin{aligned} E_f= & {} \frac{1}{2}\int _0^L\left[ C_1w_1^2+C_2w_2^2+2C_3w_1w_2\right] \,\mathrm{d}a+\left( m_f^{(1)}+m_f^{(2)}+m_f^{(3)}-\int _0^LA_1\,\mathrm{d}a\right) \frac{(p-I)^2}{2A^2}\\&+\,\int _0^L\frac{g}{2x_{1a}}\left[ \widetilde{\rho }_{[1,3]}\chi _1^2+\widetilde{\rho }_{[2,3]}\chi _2^2\phi _{2a}^2+2\widetilde{\rho }_{[2,3]}\chi _1\chi _2\phi _{2a}\right] \,\mathrm{d}a+\frac{1}{2}gd^2\rho _3L. \end{aligned}$$

The results of the linear simulation in Figs. 13 and 14 show excellent agreement with the linear solution (8.26) for the $1\mathrm{st}$ (lowest frequency) mode of the characteristic equation (8.40), given by the red dots. The error in the system energy $\widehat{\mathscr {H}}_N(t)=\mathscr {H}_N(t)-\mathscr {H}_N(0)$, for this simulation is small, $O(10^{-13})$, where $\mathscr {H}_N$ is the discretized form of the Hamiltonian (8.14). The slight increase in the error over the duration of the simulation is believed to be due to the Kelvin–Helmholtz instability, but this error growth is not large enough to affect the result and hence is tolerable.

The nonlinear results in Figs. 15 and 16 compare directly to the two-layer simulation in Figs. 8 and 9. This result is given by the red dots in Figs. 8a and 9. The comparison with the two-layer result is excellent, with the slight discrepancy in the two results at large times due to the two sets of simulation parameter values not being identical ($\rho _2=990$ kg m$^{-3}$ not 1000 kg m$^{-3}$). The energy error in Fig. 15b again grows slightly in time, due to the Kelvin–Helmholtz instability, but as it is $O(10^{-7})$, it is tolerable for the results presented.

As a final note, the growth in the energy error, which we believe is a consequence of the Kelvin-Helmholtz instability can also be observed in the two-layer simulations when $\rho _1\approx \rho _2$ through a growing energy error $\widehat{\mathscr {H}}_N(t)$, but in this case, the growth is not as obvious as in the three-layer simulations presented here.
